# A *Toxoplasma* Prolyl Hydroxylase Mediates Oxygen Stress Responses by Regulating Translation Elongation

**DOI:** 10.1128/mBio.00234-19

**Published:** 2019-03-26

**Authors:** Celia Florimond, Charlotte Cordonnier, Rahil Taujale, Hanke van der Wel, Natarajan Kannan, Christopher M. West, Ira J. Blader

**Affiliations:** a Department of Microbiology and Immunology, University at Buffalo School of Medicine, Buffalo, New York, USA; b Institute of Bioinformatics, University of Georgia, Athens, Georgia, USA; c Department of Biochemistry and Molecular Biology, University of Georgia, Athens, Georgia, USA; d Center for Tropical and Emerging Global Diseases, University of Georgia, Athens, Georgia, USA; University of Arizona

**Keywords:** host-pathogen interactions, metabolism, oxygen, protein translation

## Abstract

As the protozoan parasite Toxoplasma gondii disseminates through its host, it responds to environmental changes by altering its gene expression, metabolism, and other processes. Oxygen is one variable environmental factor, and properly adapting to changes in oxygen levels is critical to prevent the accumulation of reactive oxygen species and other cytotoxic factors. Thus, oxygen-sensing proteins are important, and among these, 2-oxoglutarate-dependent prolyl hydroxylases are highly conserved throughout evolution. *Toxoplasma* expresses two such enzymes, TgPHYa, which regulates the SCF-ubiquitin ligase complex, and TgPHYb. To characterize TgPHYb, we created a *Toxoplasma* strain that conditionally expresses TgPHYb and report that TgPHYb is required for optimal parasite growth under normal growth conditions. However, exposing TgPHYb-depleted parasites to extracellular stress leads to severe decreases in parasite invasion, which is likely due to decreased abundance of parasite adhesins. Adhesin protein abundance is reduced in TgPHYb-depleted parasites as a result of inactivation of the protein synthesis elongation factor eEF2 that is accompanied by decreased rates of translational elongation. In contrast to most other oxygen-sensing proteins that mediate cellular responses to low O_2_, TgPHYb is specifically required for parasite growth and protein synthesis at high, but not low, O_2_ tensions as well as resistance to reactive oxygen species. *In vivo*, reduced TgPHYb expression leads to lower parasite burdens in oxygen-rich tissues. Taken together, these data identify TgPHYb as a sensor of high O_2_ levels, in contrast to TgPHYa, which supports the parasite at low O_2_.

## Abstract (precis)

Because oxygen plays a key role in the growth of many organisms, cells must know how much oxygen is available. O_2_-sensing proteins are therefore critical cellular factors, and prolyl hydroxylases are the best-studied type of O_2_-sensing proteins. In general, prolyl hydroxylases trigger cellular responses to decreased oxygen availability. But, how does a cell react to high levels of oxygen? Using the protozoan parasite *Toxoplasma gondii*, we discovered a prolyl hydroxylase that allows the parasite to grow at elevated oxygen levels and does so by regulating protein synthesis. Loss of this enzyme also reduces parasite burden in oxygen-rich tissues, indicating that sensing both high and low levels of oxygen impacts the growth and physiology of *Toxoplasma*.

## INTRODUCTION

Infections with the obligate intracellular parasite Toxoplasma gondii are initiated by ingesting tissue cysts from undercooked meat or oocysts shed in feline feces. Gastric enzymes rupture the cysts, and the released parasites infect the proximal small intestine (1). This infection triggers recruitment of monocytes and dendritic cells that in turn are infected and used by *Toxoplasma* to disseminate to peripheral tissues such as the brain, lung, retina, and skeletal muscle (2). Each anatomical site has a unique environment, and the challenge that *Toxoplasma* and other pathogens face is that they must optimize their metabolism and other cellular processes to each environment so that they can grow properly.

O_2_ is among the most variable environmental factors that *Toxoplasma* encounters throughout its life cycle. Aerobic cells fine-tune their metabolism and other physiological parameters to local O_2_ levels in order to maximize ATP synthesis while limiting production of reactive oxygen species and other toxic molecules (3). O_2_ is also a cue that cells use to assess their environmental localization (4). Thus, O_2_ sensing is a critical process, and owing to their typically high *K_m_* values toward O_2_, the O_2_/α-ketoglutarate-dependent prolyl-4 hydroxylase (PHD) class of nonheme dioxygenases are well adapted to function as key cellular O_2_-sensing proteins (5, 6). In metazoans, PHDs are best known by their ability to modify the alpha subunit of the hypoxia-inducible transcription factor (HIFα), which leads to its proteasomal degradation (7, 8). Thus, under O_2_-replete conditions HIFα is prolyl hydroxylated and subsequently degraded whereas at low O_2_ HIFα is stabilized, which allows it to bind HIF-1β and activate gene expression (8–10).

O_2_ sensing in protists has been studied less. Analysis of available genomes reveals that protists express PHDs but lack HIFs (4, 11, 12). The Dictyostelium discoideum PHD (DdPHYa), which is the best-characterized protist PHD, prolyl hydroxylates the adaptor protein in the SCF ubiquitin ligase complex, DdSKP1, allowing it to be modified by a series of glycosyltransferases (13, 14). SKP1 hydroxylation/glycosylation results in conformational changes that render it receptive to SCF assembly (15). DdPHYa and the DdSKP1-modifying glycosyltransferases are conserved in other protozoans, including *Toxoplasma,* where they are important for parasite growth at low O_2_ (16–18). Here we report the identification and characterization of a second *Toxoplasma* PHD that is divergent from the PHYa family of PHDs. We named this PHD TgPHYb, and the goal of this study was to characterize its cellular functions. As opposed to TgPHYa and other PHDs that detect and trigger hypoxic stress responses, we report that TgPHYb is required for growth at high O_2_ levels by regulating translation elongation during protein synthesis.

## RESULTS

### Identification of TgPHYb as a prolyl hydroxylase conserved among apicomplexans.

**FIG 1:**
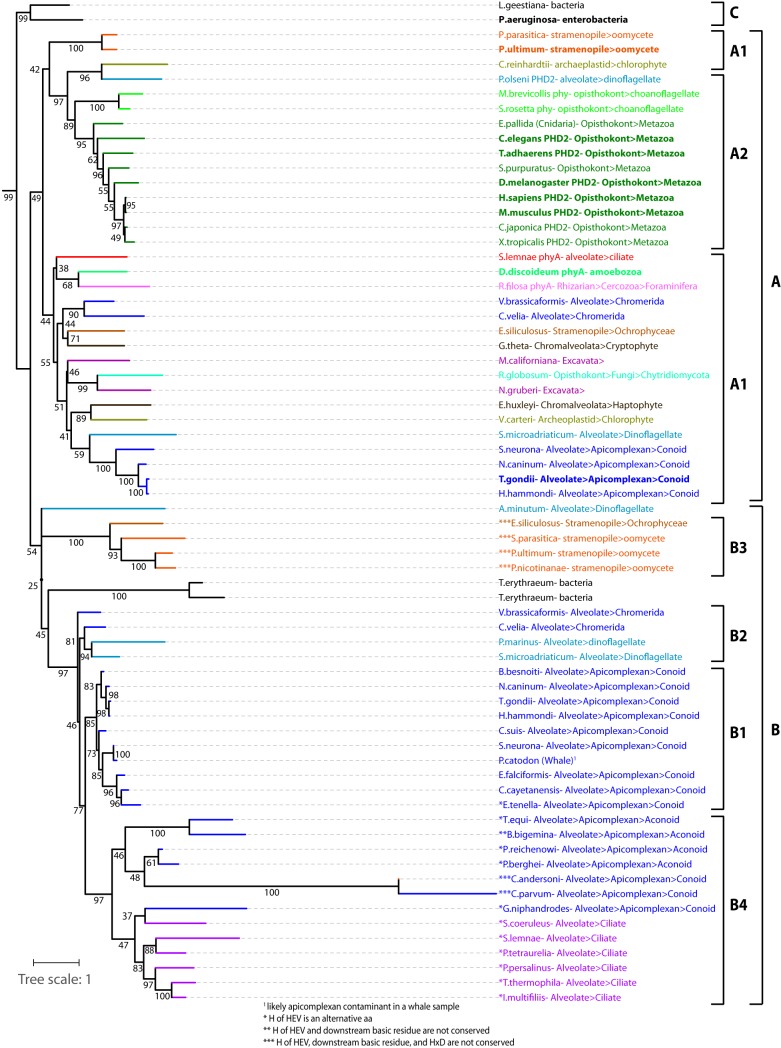
Evolutionary Origin of TgPHYb. The sequences of the catalytic domain (regions H1 and CD, Fig. S1) of all known sequences related to TgPHYb and TgPHYa were aligned (Fig. S2), trimmed of group-specific insertions, and analyzed phylogenetically using a maximum likelihood method. The structure of the tree was similar without trimming (not shown). Genes are named by the species of origin and colored according to their phylogenetic grouping. Names in bold have been experimentally validated. See Table S2 for gene IDs. Group A genes are most closely related to protist PHYas and animal PHDs, and group B genes are most closely related to TgPHYb, which resides in the B1 subclade. See text for discussion of subgroups. The tree is rooted with the bacterial EF-Tu P4H (group C). The tree scale shows the branch length that corresponds to an average number of one amino acid substitution per position, and the numbers represent bootstrap values.

A BLAST search of the *Toxoplasma* genome database (www.toxodb.org) for TgPHYa-related sequences yielded TgGT1_214620, whose transcript is predicted to comprise 11 exons encoding a 64-kDa protein. TGGT1_214620 contains a C-terminal 253-amino-acid PHD-like domain with 27% identity and 46% similarity to TgPHYa, and those amino acids considered essential for PHD catalytic activity are conserved (see Fig. S1 in the supplemental material). The evolutionary origin of TgPHYb was investigated by searching publicly accessible genome databases for and aligning the sequences of all PHYa-, PHYb-, and PHD-related genes from protists and select prokaryote and metazoan representatives (Fig. S2). Alignment of 68 predicted proteins was trimmed of species- and group-specific insertions and subjected to a phylogenetic analysis using a maximum likelihood method (Fig. 1). The tree was rooted with a clade of bacterial PHDs (group C) that prolyl hydroxylate elongation factor Tu (12, 19), because their more divergent sequences are potentially ancestral to the other genes. The sequences organize into two broad additional groups, A and B, which likely evolved from a primordial gene duplication since some species are represented in both groups. The A clade includes the protist SKP1-PHDs DdPHYa, TgPHYa, and PuPHYa (unpublished data). Since these sequences split into two subclades, it is possible that all of the sequences in the two A1 regions are Skp1 PHYas—a proposal supported by the finding that each organism also possesses a putative Gnt1 glycosyltransferase that is expected to modify the hydroxylated proline in SKP1 (20). Indeed, many PHYa genes are fused to Gnt1-like coding regions (Table S2). Each of the major protist groups are represented, as well as a fungus, indicating the presence of PhyA in the ancestral eukaryotic lineage. Each sequence conserves its catalytically important residues (Fig. S2), supporting a widespread distribution of the active enzyme. Emerging from within this clade are the metazoan PHDs that utilize HIFα rather than Skp1 as their substrates.

10.1128/mBio.00234-19.1 FIG S1 PHYb domain architecture. The H1 and CD homology domains comprise the C terminus and together represent the catalytic domain of Skp1 PhyAs and PHDs from animals. TgPHYb conserves these domains at its C terminus (Fig. S2) and in addition possesses another domain called D2 just upstream. TgPHYb has another conserved sequence further upstream, D1, that is not conserved in other TgPHYb candidates. Download FIG S1, PDF file, 0.4 MB. Copyright © 2019 Florimond et al. 2019 Florimond et al. This content is distributed under the terms of the Creative Commons Attribution 4.0 International license.

10.1128/mBio.00234-19.2 FIG S2 Alignment of PHYb-like, PHYa-like, and PHD-like sequences. All sequences related to PHYa and PHYb that could be found in publicly accessible databases (NCBI, DOE JGI, EUPATHDB, and Broad Institute) as of 1 March 2018, and representative PHD sequences, are included. See Table S2 for additional information about the sequence sources. Amino acids are color coded, positions of conservation are highlighted, and positions of perfect conservation within a group are boldfaced, as described previously (64). Subclades are separated by an underline; clades A and B are separated by a double-underline. Positions containing highly conserved residues (HxD, HxV, K/R) known to orient substrates in the active site in other P4Hs are indicated with an asterisk. In the H1 and CD domains, positions that differentiate clade B1 (and possibly nearby B subclades) sequences from clade A sequences are marked with ¶, positions that uniquely characterize the metazoan PHDs (group A2) are denoted with §, and positions that tend to characterize PHYa (A1) relative to A2 sequences are denoted with ‡. Download FIG S2, DOCX file, 0.3 MB. Copyright © 2019 Florimond et al. 2019 Florimond et al. This content is distributed under the terms of the Creative Commons Attribution 4.0 International license.

10.1128/mBio.00234-19.10 TABLE S2 List of organisms and information used for Fig. 1. Download Table S2, XLSX file, 0.02 MB. Copyright © 2019 Florimond et al. 2019 Florimond et al. This content is distributed under the terms of the Creative Commons Attribution 4.0 International license.

The remaining sequences loosely cluster in the second broad group, B (Fig. 1). TgPHYb resides in the highly conserved B1 clade, which is populated only by conoid apicomplexans. These sequences all possess the expected active site residues conserved in PHDs (Fig. S2) (12); a single exception is the sequence from Eimeria tenella, whose enzymatic activity is therefore uncertain. Several positions in the B1 clade contain invariant amino acids that are distinct from those in the PHYa clade (Fig. S2) and vice versa, confirming the significance of their belonging to separate clades. Branching slightly earlier than the clade B1 PHYb residues are sequences from chromerids (group B2), considered to be ancestral to apicomplexans (21). These are also candidates for possessing PHYb-like activity because they possess characteristic PHYb and active site residues (Fig. S2). The B2 group also contains sequences from dinoflagellates, which are distantly related to apicomplexans (22), but there is no evidence for PHYb-like sequences outside the broader alveolate group. Since a number of apicomplexans and other alveolates have both PHYb- and PHYa-like genes, PHYb likely evolved from duplication of the PHYa gene. Proteins residing in the B1 and the B2 clades are reasoned to have PHD activity because this activity is found in the last common ancestor with the bacterial EF-Tu PHDs.

The B4 clade within the B group contain relatively more divergent genes from the aconoid group of apicomplexans, which includes *Plasmodium* and *Cryptosporidium*, and from the ciliates. Remarkably, these predicted proteins lack one or more of the five critical residues (HxD, HxV, and K/R) that typify the active PHD sites (Fig. 1; Fig. S2, asterisks) and thus are potentially catalytically inactive or operate by distinct mechanisms. The fact that these genes retain some degree of mutual similarity argues for the existence of a currently unknown conserved function. However, the *Cryptosporidium* sequences appear to be rapidly evolving, suggesting an absence of purifying selection. Interestingly, a group (B3) of sequences from the stramenopiles, which are distantly related to alveolates (22), are also characterized by replacement of critical catalytic residues, reinforcing the significance of this sequence variation. Overall, B-clade sequences are characterized by a novel, conserved 120-amino-acid domain of unknown function, named the D2-domain (Fig. S1), immediately upstream of the catalytic domain. This study focuses on TgPHYb, a predicted conoid apicomplexan PHD that appears related to TgPHYa by virtue of a gene duplication in the last common ancestor of the stremopiles and alveolates within the broader SAR.

### TgPHYb is required for growth following exposure to extracellular stress.

**FIG 2:**
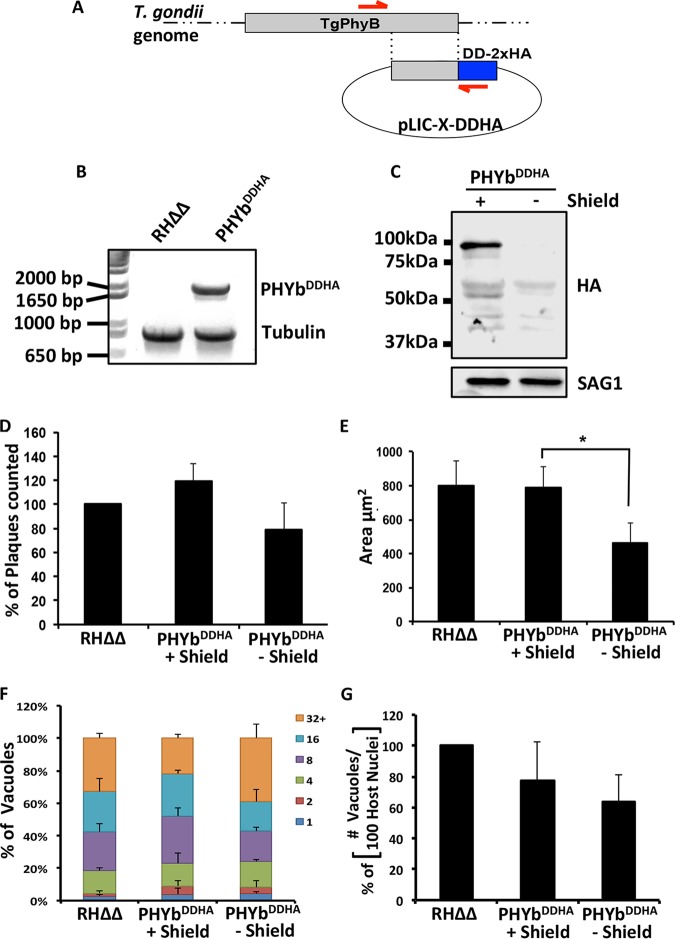
TgPHYb is required for optimal parasite growth. (A and B) Schematic diagram depicting strategy used to endogenously tag TgPHYb with DD-2×HA. Red arrows denote position of PCR primers used in panel B to confirm proper integration of the TgPHYb-DD-2×HA tag (1,935 bp). α-Tubulin (893 bp) was amplified as a positive control and coelectrophoresed with the TgPHYb amplicon. (C) Lysates from TgPHYb^DDHA^ parasites grown with or without Shield-1 for 24 h were Western blotted to detect the transgene product (using anti-HA) or SAG1 as a loading control (see Fig. S3 for lighter exposure). (D and E) Numbers (D) and areas (E) of plaques formed by freshly egressed RHΔΔ (no Shield) and TgPHYb^DDHA^ parasites grown with or without Shield-1 for 5 days. (F and G) RHΔΔ and TgPHYb^DDHA^ parasites grown with or without Shield-1 for 24 h before harvesting were added to HFFs, and 24 h later the cells were fixed. Parasite replication (F) was quantified by counting numbers of parasites per vacuole. Invasion was assessed by determining numbers of vacuoles detected per 100 host cell nuclei (G). At least 100 vacuoles per strain were counted. Shown are the averages and standard deviations from 3 independent experiments. *, *P < *0.05, Student’s *t* test.

Initial efforts to knock out TgPHYb by homologous recombination were unsuccessful, suggesting that TgPHYb is essential, which is consistent with a genome-wide CRISPR screen showing the importance of TgPHYb in parasite fitness (score = −4.6) (23). We therefore created a TgPHYb conditional expression strain (TgPHYb^DDHA^) by placing the FKBP degradation domain (DD) with a 2×HA tag at the C terminus of TgPHYb (Fig. 2A and B) (24). In the presence of the Shield-1 reagent, the DD domain is stabilized, but in its absence the domain becomes disorganized, leading to proteasomal degradation of the fusion protein. Western blotting TgPHYb^DDHA^ parasites grown with Shield-1 revealed a prominent immunoreactive band migrating at approximately 95 kDa that was undetectable when grown without Shield-1 for 24 h (Fig. 2C). Interestingly, this is ∼17 kDa higher than the hypothetical molecular weight of TgPHYb^DDHA^ (64-kDA endogenous protein + 14-kDa DD-HA tag), suggesting either that TgPHYb may be significantly posttranslationally modified by phosphorylation or other additions (25) or that its slightly high acidic amino acid content (16.6%) causes it to migrate more slowly in SDS-PAGE gels. Longer exposure of the gel revealed that a second, lower-molecular-weight band (∼80 kDa) was present specifically in the TgPHYb^DDHA^ lysate (Fig. S3) but is of unknown origin.

10.1128/mBio.00234-19.3 FIG S3 Longer exposure of Western blot in Fig. 2C. Download FIG S3, TIF file, 0.9 MB. Copyright © 2019 Florimond et al. 2019 Florimond et al. This content is distributed under the terms of the Creative Commons Attribution 4.0 International license.

To assess the importance of TgPHYb in parasite growth, freshly egressed TgPHYb^DDHA^ parasites were added to a fresh monolayer of HFFs and grown for 5 days in the absence or presence of Shield-1 (parental RHΔΔ parasites were included as a control). The monolayers were fixed, and crystal violet staining of plaques was used to assess parasite growth. Plaque numbers were similar between the samples, indicating that TgPHYb is not essential for *Toxoplasma*’s lytic growth cycle (Fig. 2D). But, plaques formed by TgPHYb^DDHA^ parasites grown without Shield-1 were significantly smaller, indicating a parasite growth defect (Fig. 2E).

*Toxoplasma* grows by a lytic cycle composed of repeated rounds of motility, host cell invasion, replication, and egress. To determine which step was affected, we used standard assays for each to assess whether one of these was affected when TgPHYb expression was reduced. The data indicated that TgPHYb depletion had no significant impact in any of these processes (Fig. 2F and G and Fig. S4).

10.1128/mBio.00234-19.4 FIG S4 TgPHYb is not required for *Toxoplasma* motility or egress. (A) Freshly harvested parasites were added to poly-L-lysine (P4707; Sigma)-coated coverslips, incubated for 15 min at 37°C, and then fixed with 3% paraformaldehyde. Adhered parasites and motility trails were detected by anti-SAG1 immunofluorescence detection. Scale bars represent 7 µm. (B) Parasites were added to HFF monolayers and 30 h later treated with DMSO (vehicle control) or A23187 to stimulate egress. Cells were fixed 3 min later, and parasites were identified by anti-SAG1 immunofluorescence detection. Percent egress was calculated by determining the number of egressed vacuoles/number of total vacuoles and multiplying by 100. Download FIG S4, TIF file, 6.9 MB. Copyright © 2019 Florimond et al. 2019 Florimond et al. This content is distributed under the terms of the Creative Commons Attribution 4.0 International license.

**FIG 3:**
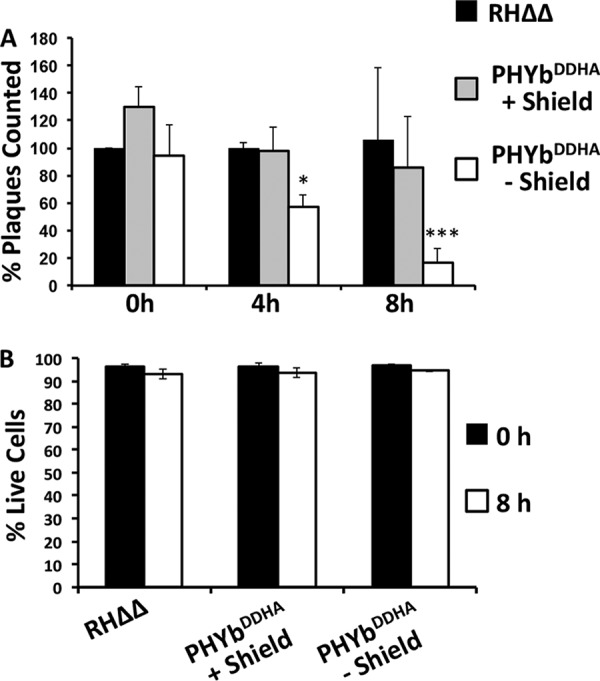
TgPHYb is required for extracellular survival. (A) TgPHYb^DDHA^ parasites grown for 24 h with or without Shield-1 were harvested, incubated extracellularly for the indicated times, and then added to HFF monolayers with or without Shield-1. Plaques were counted after 5 days and normalized to RHΔΔ parasites that were not exposed to extracellular stress. *, *P < *0.05; ***, *P < *0.001, two-way ANOVA. (B) Viability of TgPHYb^DDHA^-depleted or -replete parasites after 0 or 8 h of extracellular stress was assessed by flow cytometric analysis of vital dye staining. Shown are the averages and standard deviations from 3 independent experiments.

Besides steps in the lytic cycle, decreased plaque size may reflect changes in parasite extracellular survival (26). Thus, TgPHYb^DDHA^ parasites were grown for 24 h in the absence or presence of Shield-1, harvested, syringe lysed to liberate parasites from their host cells, and then incubated extracellularly for 0, 4, or 8 h. Parasites were then added to HFF monolayers and grown with or without Shield-1, and plaques were enumerated 5 days later. Numbers of plaques formed by TgPHYb-depleted parasites exposed to extracellular stress for 4 h were reduced by ∼40%, and this increased to 90% after 8 h (Fig. 3A). Vital dye staining indicated that decreased plaque numbers were not due to general parasite lysis induced by the extracellular stress (Fig. 3B). These data indicate that TgPHYb is required for resistance to extracellular stress.

### TgPHYb is required for growth under O_2_-replete conditions.

**FIG 4:**
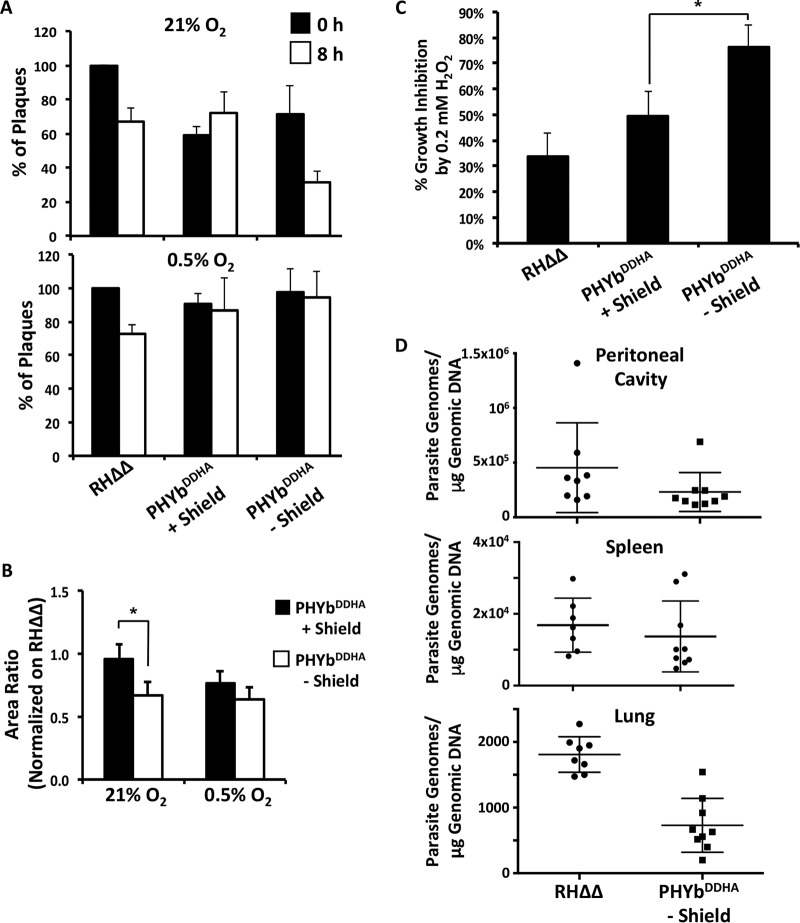
TgPHYb is not required for growth or survival at low O_2_ levels. (A and B) TgPHYb^DDHA^ parasites grown for 24 h with or without Shield-1 were harvested, exposed to stress for 0 or 8 h at 21% or 0.5% O_2_, and then added to HFF monolayers for 5 days, at which time plaque numbers (A) and area (B) were determined. (C) Equal numbers of parasites were added to HFF monolayers and grown in the absence or presence of 0.2 mM H_2_O_2_. After 5 days, monolayers were fixed, numbers of plaques were counted, and % inhibition was calculated by dividing numbers of plaques formed by parasites grown with H_2_O_2_/numbers of plaques formed by parasites grown without H_2_O_2_. *, *P* *<* 0.05, Student’s *t* test. (D) qPCR was used to measure parasite burdens in peritoneal cavities, spleens, and lungs of mice intraperitoneally infected with TgPHYb-depleted TgPHYb^DDHA^ or RHΔΔ parasites. Shown are means and standard deviations from a total of 8 to 9 mice collected from 3 independent experiments. Significant differences were observed only in lungs (*P* < 0.05, one-way ANOVA).

Since TgPHYb is a putative O_2_-sensing protein, we assessed the O_2_ dependence of TgPHYb-associated growth phenotypes. Thus, TgPHYb-replete or -depleted parasites were incubated extracellularly at 21% or 0.5% O_2_ for 0 or 8 h and added to HFFs, and plaque numbers and sizes were determined 5 days after growth at 21% O_2_. While TgPHYb-depleted parasites incubated at 21% O_2_ formed fewer and smaller plaques than did TgPHYb-replete parasites, these differences were not evident in parasites incubated extracellularly at 0.5% O_2_ (Fig. 4A and B).

Increases in O_2_ levels are often accompanied by elevated levels of reactive oxygen and nitrogen species that must be detoxified to avoid cell damage. We therefore infected host cells with freshly egressed parasites, washed the cells 2 h later to remove uninvaded parasites, and then grew the cultures for 5 days in either normal growth medium or medium containing 0.2 mM H_2_O_2_, which is approximately its IC_50_ toward *Toxoplasma* (27). As expected, 0.2 mM H_2_O_2_ reduced growth of TgPHYb-expressing parasites by approximately 50%. In contrast, growth of TgPHYb-depleted parasites was reduced by >80% (Fig. 4C).

In an infected host, *Toxoplasma* tachyzoites disseminate to tissues with a variety of O_2_ tensions. We therefore tested whether TgPHYb was required for colonization of and/or growth within O_2_-rich tissues by intraperitoneally infecting mice with freshly harvested wild-type RHΔΔ tachyzoites or TgPHYb^DDHA^ tachyzoites that had been grown for 24 h in the absence of Shield-1. After 6 days, mice were euthanized and parasite numbers were determined by PCR detection of genomic DNA in the peritoneal cavity (site of infection), spleen (low-O_2_ tissue), and lung (high-O_2_ tissue). While no significant differences were observed either in spleens or peritoneal cavities, significantly fewer TgPHYb-depleted than TgPHYb-replete parasites were present in lungs (Fig. 4D). Taken together, these data indicate that TgPHYb is important for *Toxoplasma* tachyzoites to adapt to elevated O_2_ levels.

### TgPHYb-depleted parasites display host cell invasion defects following extracellular stress.

**FIG 5:**
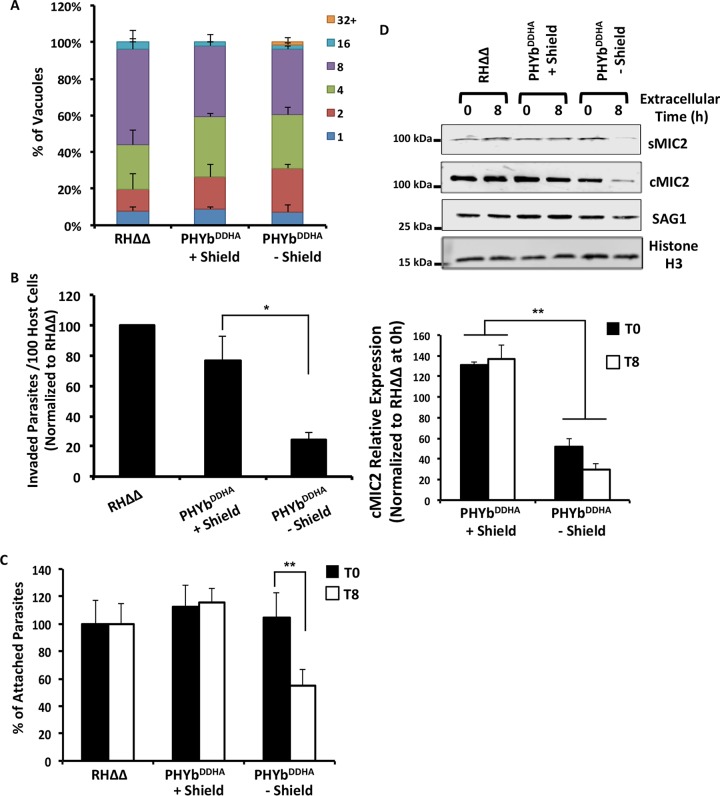
Extracellular stress leads to reduced host cell invasion by TgPHYb-depleted parasites. TgPHYb^DDHA^ parasites grown for 24 h with or without Shield-1 were harvested and exposed to stress for 0 or 8 h at 21% O_2_. (A) Replication of extracellularly stressed parasites was determined by counting numbers of parasites per vacuole 24 h postinfection. At least 100 vacuoles per strain were counted, and shown are the averages and standard deviations from three independent assays. (B) Parasites were added to HFF monolayers in high-K^+^ buffer for 20 min and replaced with prewarmed invasion medium for 1 h. The cells were fixed, and numbers of intracellular parasites were determined by differential SAG1 staining and normalized to RHΔΔ parasites. *, *P < *0.05, Student’s *t* test. (C) Parasites were added to HFF monolayers in high-K^+^ buffer for 20 min. The cells were then washed to dislodge weakly associated parasites, fixed, and stained with SAG1 antisera to determine numbers of intimately attached parasites at each time point, normalized to parental RHΔΔ strain parasites. **, *P < *0.005, Student’s *t* test. (D) Parasites were incubated with 1% (vol/vol) ethanol for 5 min to stimulate microneme discharge. After centrifugation, supernatants and parasites (from equal cell numbers) were separated by SDS-PAGE and Western blotted to detect secreted MIC2 (sMIC2), cell-associated MIC2 (cMIC2), SAG1, and *Toxoplasma* histone H3 as a loading control. Shown are the average and standard deviations from 3 independent experiments as well as representative blots.

We next sought to determine which step(s) of the lytic cycle was affected when TgPHYb-depleted parasites were exposed to extracellular stress. To assess replication, TgPHYb-replete or -depleted parasites were incubated extracellularly for 8 h, added to HFFs, and fixed 24 h later. The cells were then stained to detect the parasites by immunofluorescence microscopy, and numbers of parasites per vacuole were determined. The data revealed that changes in TgPHYb expression levels did not impact *Toxoplasma* replication rates following extracellular stress (Fig. 5A).

We did note, however, that fewer numbers of vacuoles formed in cells infected with extracellularly stressed TgPHYb-depleted parasites. Since Fig. 3B showed that TgPHYb depletion did not reduce viability, these data suggested that depletion reduced parasite invasion following extracellular stress. To directly test this, parasites were incubated extracellularly for 8 h and then added to HFFs in high-potassium buffer that permits parasite attachment to, but not invasion of, host cells (28). Invasion buffer was then gently added to trigger invasion, and 20 min later, the cells were directly fixed and numbers of intracellular and extracellular parasites were determined. TgPHYb depletion significantly reduced invasion of extracellularly stressed TgPHYb-depleted parasites (Fig. 5B).

A critical step in host cell invasion is formation of an intimate contact between the parasite and host cell that is resistant to vigorous washing (29). To test whether decreased TgPHYb expression affected intimate attachment, invasion assays were performed as described above, but before fixation the monolayers were vigorously washed with ice-cold buffer to remove loosely associated parasites while preventing invasion from proceeding. Decreased TgPHYb expression led to reduced intimate contact formation following extracellular stress (Fig. 5C).

Intimate host cell attachment is mediated by parasite adhesins that are released from specialized secretory organelles named micronemes (29). During invasion, microneme secretion is triggered by elevating intracellular calcium levels, which can be artificially stimulated by ethanol (30). Thus, TgPHYb-replete or -depleted parasites were incubated extracellularly for 0 or 8 h, and then 1% ethanol was added to trigger microneme secretion. Equivalent fractions of culture supernatants and cell pellets were separated by SDS-PAGE and Western blotted to detect the transmembrane micronemal protein MIC2, which after its exocytosis is proteolytically released into the culture supernatant by a rhomboid protease (31–33). Significantly reduced amounts of MIC2 (sMIC2) were released by extracellularly stressed (8 h) TgPHYb-depleted parasites (Fig. 5D). We also noted that levels of cell-associated MIC2 (cMIC2) and the SAG1 surface protein were reduced but not those of histone H3, which was therefore used as a loading control.

### TgPHYb regulates translation elongation during extracellular stress.

**FIG 6:**
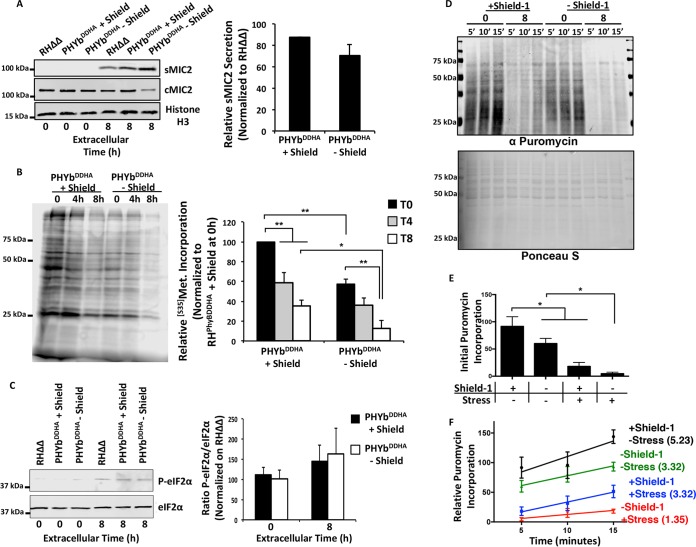
TgPHYb is required for *de novo* protein synthesis during extracellular stress. TgPHYb^DDHA^ parasites grown for 24 h with or without Shield-1 were harvested and exposed to stress for 0, 4, or 8 h at 21% O_2_. (A) Parasites were pelleted by centrifugation. MIC2 was detected in equivalent volumes of the pelleted parasites (cMIC2) and supernatants (sMIC2) collected from the parasites following 8 h of incubation. *Toxoplasma* histone H3 was used as a loading control. Shown are the average and standard deviations from 3 independent experiments as well as representative blots. (B) Autoradiograph of parasites pulse-labeled with [^35^S]Met-Cys for 1 h following exposure to extracellular stress for 0, 3, or 7 h. Shown are the average and standard deviations from 3 independent experiments as well as representative blots. *, *P < *0.05; **, *P < *0.005, one-way ANOVA. (C) Western blot detection of phospho-eIF2α or total eIF2α in parasites exposed to extracellular stress for 0 or 8 h. Shown are the average and standard deviations from 3 independent experiments as well as representative blots. A Student *t* test indicated that differences were not statistically significant. (D) Freshly egressed parasites or parasites incubated extracellularly for 8 h were incubated with puromycin for the indicated times. Lysates were prepared, separated by SDS-PAGE, and then either Western blotted to detect puromycylated peptides (top) or stained with Ponceau S (bottom). Shown are representative blots from 3 independent experiments. (E) Initial puromycin incorporation levels were determined by ratios of puromycin to Ponceau S staining intensities after 5 min of adding puromycin. Data were normalized to unstressed parasites in the presence of Shield-1. Shown are averages and standard deviations from 3 independent experiments. *, *P < *0.05, Student’s *t* test. (F) Puromycin/Ponceau S labeling intensities were determined for each time point, and the slope (shown as number on label) of each line was calculated to determine rate of puromycin incorporation.

Extracellularly incubated tachyzoites spontaneously exocytose low levels of micronemal proteins (34, 35). Thus, reduced secreted and cell-associated MIC2 levels could be due to an inability to replenish the MIC2 and likely other micronemal proteins that were secreted during the 8-h incubation period. We first tested this hypothesis by confirming that MIC2 was indeed released during the extracellular incubation (Fig. 6A) without a change in its transcript levels (Fig. S5).

10.1128/mBio.00234-19.5 FIG S5 TgPHYb does not regulate MIC2 transcript levels. qRT-PCR quantification of MIC2 and ROP18 transcript levels from parasites that were incubated extracellularly for 0 or 8 h. Download FIG S5, TIF file, 3.1 MB. Copyright © 2019 Florimond et al. 2019 Florimond et al. This content is distributed under the terms of the Creative Commons Attribution 4.0 International license.

mRNA translation in tachyzoites is reduced upon exposure to prolonged extracellular stress due to a block in translation initiation (26). To test whether loss of TgPHYb exacerbated this protein synthesis defect, we first compared the protein synthesis capacities of TgPHYb-replete and -depleted parasites by [^35^S]methinone labeling. Consistent with earlier work, extracellular stress reduced ^35^S-labeled amino acid incorporation by wild-type parasites in a time-dependent manner (Fig. 6B). Strikingly, ^35^S labeling of TgPHYb-depleted parasites was significantly decreased in the absence of extracellular stress, and this decrease was enhanced in parasites that were incubated extracellularly for 8 h (Fig. 6B).

Extracellular stress reduces mRNA translation by promoting phosphorylation of the eukaryotic initiation factor complex protein eIF2α. Because eIF2α is inhibited when it is phosphorylated, we compared eIF2α phosphorylation status between TgPHYb-replete or -depleted parasites that were either freshly egressed or incubated extracellularly for 8 h. eIF2α did not appear to be phosphorylated in freshly egressed replete or depleted parasites. In contrast, eIF2α phosphorylation was increased in tachyzoites that were incubated extracellularly for 8 h, consistent with a previous study (26). But, eIF2α phosphorylation was not further increased by TgPHYb depletion (Fig. 6C).

The lack of an effect on eIF2α phosphorylation as well as decreased [^35^S]methionine labeling of protein synthesis in TgPHYb-depleted parasites suggested that another process during mRNA translation was downstream of TgPHYb. Puromycin is an aminoacyl-tRNA mimetic that becomes covalently attached to nascent peptides and in conjunction with an antipuromycin antibody can be used to assess rates of protein elongation (36, 37). Thus, puromycin was added to freshly egressed or extracellularly stressed TgPHYb-replete or -depleted parasites, and lysates were prepared 5, 10, or 15 min later and Western blotted to detect puromycylated peptides (Fig. 6D). Initial (5-min) puromycin labeling was significantly reduced in TgPHYb-depleted freshly egressed parasites (Fig. 6E), which is consistent with the ^35^S-labeling experiments (compare zero time points in Fig. 6B). Moreover, initial puromycin labeling was dramatically reduced in the stressed TgPHYb-replete parasites by ∼75%, which was likely due to decreased translation initiation (26), and was further reduced by >90% in the TgPHYb-depleted parasites.

Next, elongation rates were determined by calculating the slopes of the line for each condition (Fig. 6F). Puromycin incorporation rates were similar between freshly egressed and stressed TgPHYb-expressing parasites, as well as freshly egressed TgPHYb-depleted parasites. In contrast, rates of puromycin incorporation were lower when TgPHYb-depleted parasites were exposed to extracellular stress. These effects were not due to unanticipated effects of the Shield-1 reagent on wild-type parasites, since puromycin incorporation was unaffected in parental RHΔΔ parasites treated with or without Shield-1 (Fig. S6).

10.1128/mBio.00234-19.6 FIG S6 Shield-1 does not affect puromycin incorporation. (A) Puromycin incorporation assay of fresh parasites grown in the absence or presence of Shield-1 for 24 h and then incubated extracellularly with puromycin in the absence or presence of Shield-1. Ponceau staining was used as a loading control. (B) Graph of puromycin incorporation rates. The slope of each line represents puromycin incorporation rates, and no significant differences were found between RHΔΔ without Shield-1 (3.25 ± 0.6813) and RHΔΔ with Shield-1 (2.76 ± 0.993). Shown are the average and standard deviations from 3 independent experiments as well as representative blots. Download FIG S6, TIF file, 8.8 MB. Copyright © 2019 Florimond et al. 2019 Florimond et al. This content is distributed under the terms of the Creative Commons Attribution 4.0 International license.

**FIG 7:**
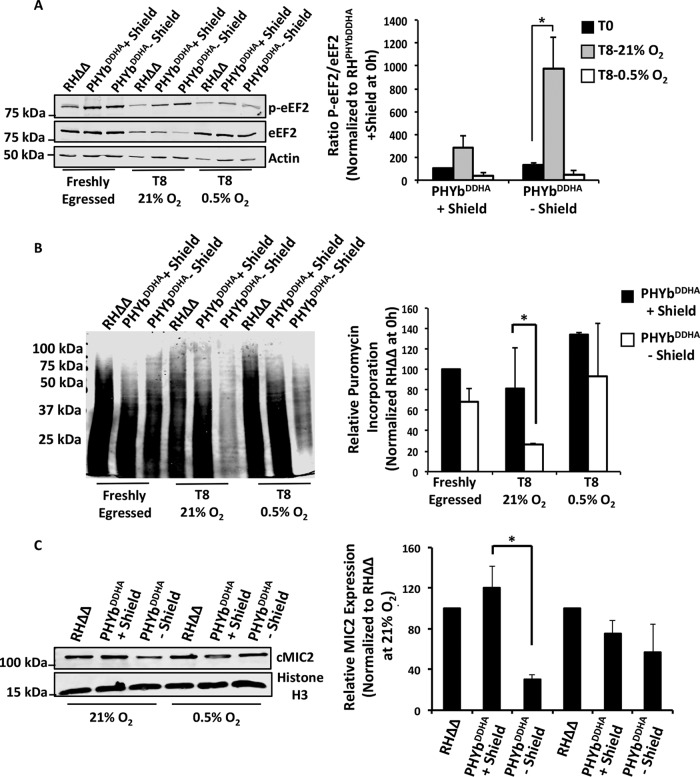
TgPHYb regulates translation elongation specifically at elevated O_2_ levels. (A) Lysates from freshly egressed parasites or parasites extracellularly stressed at 21% or 0.5% O_2_ were Western blotted to detect eEF2 and phosphorylated eEF2. Shown are the average and standard deviations from 3 independent experiments as well as representative blots. (B) Puromycylated peptides were detected by Western blotting lysates prepared from either freshly egressed parasites or parasites incubated extracellularly for 8 h at 21% O_2_ or 0.5% O_2_, which were incubated with puromycin for 30 min. Shown are the average and standard deviations from 3 independent experiments as well as representative blots. (C) Cell-associated MIC2 (cMIC2) levels were compared between parasites extracellularly stressed at 21% and 0.5% O_2_. Shown are the average and standard deviations from 3 independent experiments as well as representative blots. *, *P* < 0.05, Student’s *t* test.

Translocation of an emerging peptidyl-tRNA from the A to P site of the ribosome requires eukaryotic elongation factor 2 (eEF2), and phosphorylation of this GTPase inhibits elongation (38, 39). To address the involvement of eEF2 in stressed cells, lysates from TgPHYb-replete or -depleted parasites incubated at 21% O_2_ were Western blotted with antibodies that recognize either eEF2 or phosphorylated eEF2. In contrast to eIF2α, whose increased phosphorylation was not TgPHYb dependent, eEF2 phosphorylation was specifically increased in TgPHYb-depleted parasites (Fig. 7A). We also noted consistently lower levels of total eEF2 protein in parasites with reduced TgPHYb expression. Taken together, these data indicate that decreased TgPHYb expression reduces elongation during mRNA translation by inhibiting eEF2 activity through increased phosphorylation as well as decreased expression levels.

The rescue of the parasite growth and extracellular stress phenotypes of TgPHYb-depleted parasites at low O_2_ suggested that defects in translation elongation would be reversed at 0.5% O_2_. This was first tested by examining puromycylation and eEF2 phosphorylation in parasites incubated at this O_2_ tension. Thus, TgPHYb-replete or -depleted parasites were exposed to extracellular stress for 0 h (freshly egressed) or 7.5 h (T8) at 21% or 0.5% O_2_ and then puromycin was added for 30 min. As shown in Fig. 7B and Fig. S7, relative to 21% O_2_, puromycin incorporation was significantly increased when parasites were incubated extracellularly at 0.5% O_2_. In addition, the polypeptides observed in the parasites incubated at low O_2_ were larger than the labeling observed at 21% O_2_, indicating more efficient elongation. This correlated with reduced phosphorylation and increased expression of eEF2 in TgPHYb-depleted parasites at 0.5% O_2_ (Fig. 7A). Importantly, TgPHYb abundance was not apparently affected by extracellular stress or O_2_ exposure (Fig. S8). Finally, MIC2 expression levels were restored in TgPHYb-depleted parasites incubated extracellularly at 0.5% O_2_ (Fig. 7C), further supporting the hypothesis that loss of TgPHYb impacts translation elongation and protein synthesis specifically at elevated O_2_ levels.

10.1128/mBio.00234-19.7 FIG S7 Shorter exposure of Western blot in Fig. 7B. Download FIG S7, TIF file, 2.7 MB. Copyright © 2019 Florimond et al. 2019 Florimond et al. This content is distributed under the terms of the Creative Commons Attribution 4.0 International license.

10.1128/mBio.00234-19.8 FIG S8 TgPHYb expression levels under extracellular and O_2_ stress. Freshly egressed (T0) TgPHYb^DDHA^ parasites were incubated extracellularly at 21% or 0.5% O_2_ for 8 h. Lysates were prepared and Western blotted to detect HA-tagged TgPHYb (αHA) or SAG1 (as a loading control). Download FIG S8, TIF file, 2.7 MB. Copyright © 2019 Florimond et al. 2019 Florimond et al. This content is distributed under the terms of the Creative Commons Attribution 4.0 International license.

## DISCUSSION

PHDs have emerged as critical O_2_-sensing proteins because their higher *K_m_* values toward O_2_ allow their enzymatic activity to be regulated over a broad range of physiological O_2_ tensions (7, 40). Rate-limiting O_2_ levels lead to an accumulation of unmodified substrate protein enabling the cell to respond to the decreased O_2_ levels. Besides hypoxia, cells must also detect and respond to O_2_ levels that are above a reference baseline, to limit ROS production and cytotoxicity (41) or for positional information. During hyperoxic stress, PHDs remain active and therefore have traditionally been thought to have a limited function in this regime. Thus, cells have largely been thought to detect and respond to hyperoxic stress through PHD-independent mechanisms. Our discovery that a *Toxoplasma* PHD, TgPHYb, is required for resistance to hyperoxic stress therefore represents a novel function for the PHDs.

As orally transmitted pathogens, *Toxoplasma* bradyzoites or sporozoites first infect enterocytes of the small intestine, which is relatively low in O_2_. From there, they convert into tachyzoites and then disseminate to other tissues such as muscle, lung, and brain, whose O_2_ tensions can vary widely (even within the same tissue) due to a variety of factors, including distance from the vasculature (42, 43). We previously reported that TgPHYa is important for tachyzoite growth at low O_2_ tensions (16) and here show that TgPHYb is required for adaptation to higher O_2_ levels *in vitro* as well as being important for tachyzoite infection/colonization of an O_2_-rich tissue, such as the lung. TgPHYb is encoded by the TgGT1_214620 gene, and like TgPHYa (TgGT1_232960), its expression is similar between different parasite strains and life stages (http://toxodb.org/toxo/app/record/gene/TGGT1_214620). Thus, we hypothesize that the 2 prolyl hydroxylases act in concert to allow *Toxoplasma* tachyzoites to grow in a diverse array of anatomical sites and niches. We have yet to assess whether TgPHYb plays a role in cyst development and parasite persistence, since the RH strain used to generate TgPHYb^DDHA^ does not readily form cysts *in vitro* and kills mice before cysts can develop *in vivo*. Future studies using cystogenic strains are therefore needed, but based on preliminary work using a TgPHYa mutant type II strain, we expect that O_2_-sensing proteins will have complex roles in chronic infections (C. Cordonnier and I. Blader, unpublished data). It is also interesting that besides not being able to form tissue cysts, RH strain parasites survive extracellularly better than other strains (44). While the basis for this difference remains unclear, it is intriguing that other *Toxoplasma* strains express TgPHYb transcripts at significantly lower levels than RH strain parasites do (http://toxodb.org/toxo/app/record/gene/TGGT1_214620#ExpressionGraphs). Thus, TgPHYb may be a critical and rate-limiting factor in allowing a tachyzoite to survive outside its intracellular niche.

mRNA translation consists of three major steps—initiation, elongation, and termination. Extracellular stress reduces *Toxoplasma* protein synthesis by increasing eIF2α phosphorylation that subsequently reduces translation initiation by preventing ribosome binding to tRNA^Met^ (26). eIF2α phosphorylation was not impacted by TgPHYb expression levels or by O_2_ availability during extracellular stress. On the other hand, decreased TgPHYb protein at 21% O_2_ levels led to increased eEF2 phosphorylation as well as decreased rates of puromycin incorporation in stressed parasites. Together, these data suggest that mRNA translation is a key stress response pathway that *Toxoplasma* controls by regulating both initiation and elongation. Our data also reveal that mRNA translation regulation during extracellular stress is complex. First, defects in translation elongation were reversed at low O_2_, revealing that sensing of environmental cues impacts mRNA translation rates. Second, the abundances of individual proteins were differentially affected by stress: MIC2, SAG1, and eEF2 were downregulated while eIF2α and histone H3 were unaffected. These differences may be due to differences in each protein’s half-life or altered translational state. Alternatively, some proteins may be released via secretion or shedding or in exosomes during extracellular stress, and TgPHYb regulation of translation elongation is necessary to ensure that these proteins are replenished so that the parasite remains competent for invasion. Discriminating between these possibilities, which are not mutually exclusive, is critical because elongation has emerged as a drug target in apicomplexan parasites (45).

In contrast to *Toxoplasma,* where translation elongation is inhibited at higher O_2_ levels, mammalian eEF2 is phosphorylated when cells are exposed to hypoxia and this is due to prolyl hydroxylation of eEF2 kinase (46, 47). In addition, the bacterial eEF2 homologue, eEF-Tu, is a substrate of an oxygen-dependent PHD, although the impact of this modification on translation elongation remains to be determined. Based on these data, we hypothesize that either eEF2 itself or an as-of-yet-uncharacterized *Toxoplasma* eEF2 kinase is likely to serve as the substrate for the TgPHYb prolyl hydroxylation. Substrate identification is important not only to know how TgPHYb controls translation elongation but also because kinetic characterization of TgPHYb requires a substrate for the reaction.

But what role does extracellular stress play in *Toxoplasma* pathophysiology? As an obligate intracellular parasite, one would initially suspect extracellular stress responses would not play critical roles in *Toxoplasma* growth. However, in an actively infected tissue ∼20% of tachyzoites are found to be extracellular (48), and these parasites are exposed to much harsher conditions than when incubated *in vitro* in the absence of host cells. For example, extracellular parasites *in vivo* are likely to be engaged by neutrophils and macrophages that release antimicrobial factors such as peptides and reactive oxygen species (ROS), which we showed is able to more efficiently kill TgPHYb-depleted parasites. While ROS is generated under both hypoxic and normoxic conditions, the specific site where ROS is synthesized may be important in dictating cellular responses to ROS. For example, ROS generated by mitochondrial complex III is necessary and sufficient to activate HIF-1 whereas cytoplasmically generated ROS cannot (49, 50).

In summary, we have identified a novel O_2_-sensing prolyl hydroxylase required for survival under O_2_-replete conditions. This physiological role is reminiscent of DdPhyA, the Skp1 PHD from *Dictyostelium*, which has a high *K_m_* for O_2_ that supports detection of high levels of environmental O_2_ required for sporulation (13, 14, 51). This contrasts with the very low O_2_ *K_m_* of the orthologous *Toxoplasma* Skp1 PHD (16), TgPHYa, thus generating the opportunity for TgPHYb to evolve to function in higher-O_2_ niches. The second enzyme is required for optimal parasite growth in high-O_2_ environments both *in vitro* and *in vivo* and, given its limited conservation beyond the apicomplexan family of protozoans, may serve as a novel drug target.

## MATERIALS AND METHODS

### Ethics.

Animal protocols (MIC12093Y) were approved by the University at Buffalo’s IACUC and carried out in accordance with Public Health Service policy on the humane care and use of laboratory animals and AAALAC accreditation guidelines.

### Phylogenetic analyses.

Sequences related to TgPHYb were analyzed as described previously (18). In brief, the initial hit from *Toxoplasma* (TGGT1_214620) was used as query for BLAST searches against sequence databases (NCBI, DOE JGI, EUPATHDB, and Broad Institute). The best hit sequences were then collected and aligned using the MEGA alignment followed by manual curation to correct misaligned regions and trim highly gapped insert regions. The phylogenetic tree was built using IQ-Tree (52) with the following options: -m MFP -bb 10000. ModelFinder (53), implemented in IQ-Tree, was used to find the best-fit model. Bootstrap support values were obtained using UltraFast bootstrap approximation (UFBoot) (54) implemented in IQ-Tree. The overall topology of the tree was robust, as independent trees generated using RAxML (55) and FastTree (56) with either trimmed or untrimmed alignments had similar topologies.

### Cells and parasites.

The *Toxoplasma gondii* type I strain RH*ΔhxgprtΔku80* (RHΔΔ) (gift from David Bzik, Dartmouth University) and other strains generated here were maintained by passage in human foreskin fibroblasts (HFFs) (from American Type Tissue Culture, Reston, VA) in complete medium (Dulbecco’s modified Eagle medium supplemented with 10% heat-inactivated fetal calf serum, l-glutamine, and penicillin-streptomycin) as previously described (57). TgPHYb^DDHA^ (see Fig. S4 in the supplemental material) was created by ligation-independent cloning as described in reference 58. Briefly, 1 kb upstream from the predicted *TgPHYb* stop codon was PCR amplified using the primers shown in Fig. 2A and listed in Table S1. The amplicon was purified and cloned into pLIC-DD-2xHA (from Vern Carruthers, University of Michigan). The plasmid was linearized with SgrAI, transfected into RHΔΔ, and transfectants were cloned by pyrimethamine selection in the presence of 0.5 µM Shield-1 (CheminPharma, Branford, CT). Isolated clones were screened by PCR and Western blotting.

10.1128/mBio.00234-19.9 TABLE S1 List of primers and antibodies used for this study. Download Table S1, PDF file, 0.1 MB. Copyright © 2019 Florimond et al. 2019 Florimond et al. This content is distributed under the terms of the Creative Commons Attribution 4.0 International license.

### Parasite phenotype assays.

For all assays, parasites harvested from unlysed HFF monolayers were released by syringe lysis by passage through a 27-gauge needle followed by washing in serum-free medium. Parasite plaquing, synchronized invasion and attachment, motility, and replication assays were performed as described previously (59, 60). Extracellular stress was applied by incubating the parasites in complete medium at 37°C at either 21% O_2_ or 0.5% O_2_ (Invivo2 Baker, Sanford, ME). Egress assays were performed essentially as described previously (61). Briefly, parasites were grown in HFFs for 30 h and then the calcium ionophore A23187 (8 µM) (Calbiochem, Temecula, CA) was added for 3 min at 37°C. Cells were fixed, stained to detect SAG1, and then imaged by fluorescence microscopy. At least 25 vacuoles were counted per condition; collapsed vacuoles surrounded by extracellular parasites were considered egressed.

### Viability assay.

Extracellular parasites were collected by centrifugation and resuspended in fixable viability stain 510 (BD Biosciences) for 15 min at room temperature. They were then washed in 1% bovine serum albumin in phosphate-buffered saline (0.01 M sodium phosphate buffer, pH 7.4, 0.15 M NaCl) (PBS), fixed in 2% paraformaldehyde in PBS for 10 min at room temperature, and then analyzed by flow cytometry on a FACSCalibur cytometer (Becton, Dickinson). Heat-killed parasites were obtained after incubation at 56°C for 10 min and used as a positive control.

### *Toxoplasma* tissue burden.

Freshly harvested TgPHYb^DDHA^ (grown for 24 h in the absence of Shield-1) or RHΔΔ parasites at 10^3^ were injected intraperitoneally into C57BL/6J mice (Jackson Laboratory, Bar Harbor, ME). After 6 days, mice were sacrificed; peritoneal cavity exudate, spleen, and lungs were harvested and weighed; and gDNA was isolated using the DNA tissue kit (Omega, Norcross, GA). gDNA (100 ng) was mixed with *Toxoplasma* B1 primers and analyzed by real-time qPCR as described previously (62). Numbers of parasites were calculated from a standard curve generated in parallel using purified RHΔΔ DNA.

### Microneme secretion.

Microneme secretion assays were performed essentially as described previously (30). Briefly, ethanol was added (final concentration, 1% [vol/vol]) to extracellular parasites (2 × 10^6^) in complete medium, which were then incubated for 5 min at 37°C and placed on ice for 5 min. After centrifugation at 2,000 × *g* for 8 min, the supernatant (excreted secreted antigens [ESAs]) and pellet fractions were resuspended in sample buffer prior to analysis by Western blotting (see below).

### Western blotting.

Parasites were pelleted by centrifugation at 2,000 × *g* for 8 min at 4°C and lysed in boiling SDS-PAGE sample buffer containing 5% β-mercaptoethanol. Lysates from equivalent cell numbers were separated by SDS-PAGE and transferred to nitrocellulose membranes, which were blocked with Odyssey blocking buffer (Li-Cor Biosciences, Lincoln, NE), incubated with antibodies listed in Table S1, imaged using a Li-Cor Odyssey scanner, and analyzed using Image Studio software (Li-Cor).

### qRT-PCR.

qRT-PCR was performed as described previously and calculated using the 2^−ΔΔ^*^CT^* method (63). Briefly, total RNA was DNase treated and converted to cDNA using random hexamers. The cDNA was added to gene-specific primers (see Table S1) and SYBR green master mix (Thermo Fisher Scientific) and then run and analyzed using an ABI 7500 Fast RT-PCR machine (Applied Biosystems).

### [^35^S]methionine incorporation.

Freshly harvested parasites were resuspended in completed DMEM at 37°C for 0, 4, or 8 h before cells were washed in PBS and resuspended in methionine/cysteine-free DMEM supplemented with 5% dialyzed FBS, 1 mM glutamine, 0.5 mM sodium pyruvate, and 145 μCi Express Mix [^35^S]Met-Cys (Perkin Elmer, Massachusetts) for 1 h at 37°C (21% O_2_, 5% CO_2_). After washing twice with PBS, cell extracts were prepared and equivalent cell numbers (3 × 10^6^) were solubilized in Laemmli sample buffer with 2-mercaptoethanol and separated by SDS-PAGE. Radioactivity was detected in dried gels using a Typhoon Imager (GE Biosciences, Pittsburgh, PA). Each full lane was quantified densitometrically, and data were normalized to unstressed TgPHYb-replete parasites.

### Translation elongation assay.

Puromycin incorporation was performed essentially as described previously (37). Freshly harvested parasites (3 × 10^6^) were resuspended in 1 ml of complete DMEM and incubated for 0 or 8 h at 37°C under an atmosphere of 21% or 0.5% O_2_. Puromycin (10 μg/ml) (Sigma, St. Louis, MO) was added, and incubations continued at 21% or 0.5% O_2_ at 37°C. Parasites were then pelleted, washed in ice-cold PBS, and analyzed by Western blotting using an antipuromycin antibody (Table S1). Each lane was scanned and quantified densitometrically. Puromycin incorporation was normalized to the Ponceau S stain intensity of the membranes, and translation rates were calculated from slopes of the lines generated by linear regression analysis with Prism software (GraphPad, La Jolla, CA) (36). Experiments were performed three independent times.

### Statistics.

Unless otherwise noted, all experiments were repeated a minimum of three times. Data were expressed as mean ± SD and plotted, and statistical significance was determined with Prism software (GraphPad, La Jolla, CA) using appropriate assays.
