# Supplementary material for: A Toxoplasma Prolyl Hydroxylase Mediates Oxygen Stress Responses by Regulating Translation Elongation
Source: mBio. 2019 Mar 26;10(2):e00234-19. doi: 10.1128/mBio.00234-19 (PMC6437050; doi:10.1128/mBio.00234-19)
Supplement: FIG S1 [file mBio.00234-19-sf001.pdf]

Figure S1

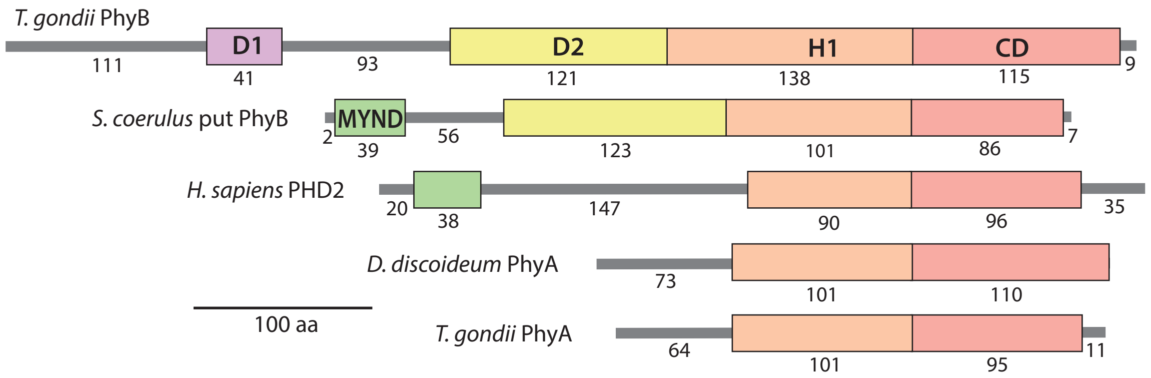

Supplemental Figure S1. PhyB domain architecture.

The H1 and CD homology domains comprise the C-terminus and together represent the catalytic domain of Skp1 PhyA's and PHDs from animals. TgPHYb conserves these domains at its C-terminus (Fig. S2), and in addition possesses another domain called B2 just upstream. TgPHYb has another conserved sequence further upstream, B1, that is not conserved in other TgPHYb candidates.
