# Supplementary material for: A Toxoplasma Prolyl Hydroxylase Mediates Oxygen Stress Responses by Regulating Translation Elongation
Source: mBio. 2019 Mar 26;10(2):e00234-19. doi: 10.1128/mBio.00234-19 (PMC6437050; doi:10.1128/mBio.00234-19)
Supplement: FIG S2 [file mBio.00234-19-sf002.docx]

# Figure S2. Alignment of PhyA-, PhyB- and PHD2-like sequences

**Group Symbol Genus Species Gene ID Phylum**

C Pa *Pseudomonas aerigunosa* AAG03699.1 proteobacteria

C Lg *Legionella geestiana* WP_028386860.1 bacteria

A1 Pp *Phytophthora parasitica* XP_008901361.1 Stramenopile

A1 PuA *Pythium ultimum* PYU1_T004073 Stramenopile

A1 Cr *Chlamydomonas reinhardtii* jgi|Chlre3|168638| Chromalveolata

A1 SlA *Stylonychia lemnae* Contig17973:1..4411 Alveolata

A1 Dd *Dictyostelium discoideum* DDB_G0277759 Amoebazoans

A1 Rf *Reticulomyxa filosa* GI:569353547 Rhizaria

A1 VbA *Vitrella brassicaformis* CEM16584.1 Alveolata

A1 CvA *Chromera velia* CVEL_22358 Alveolata

A1 EsA *Ectocarpus siliculosis* GI:298707772 Alveolata

A1 Gt *Guillardia theta* GI:428176508 Chromalveolata

A1 Rg *Rhizoclosmatium globosum* MCGO01000206.1 fungi

A1 Ng *Neagleria gruberi* in contig 20 of scaffold 19 Excavate

A1 Mc *Malawimonas californiana* EC716818.1 Excavate

A1 Eh *Emiliania Huxleyi* XP_005760621.1 Chromalveolata

A1 Vc *Volvox carteri* XP_002955656.1 Chromalveolata

A1 SmA *Symbodinium microadriaticum* OLP91867.1 Alveolata

A1 SnA *Sarcocystis neurona* SRCN_6476 Alveolata

A1 NcA *Neospora caninum* NCLIV_032800 Alveolata

A1 TgA *Toxoplasma gondii* TGME49_232960 Alveolata

A1 HhA *Hammondia hammondi* HHA_232960 Alveolata

A2 Po *Perkinsus olseni* EU007818.1 Alveolata

A2 Mb *Monosiga brevicollis* XP_001748412.1 Excavate

A2 Sr *Salpingoeca rosetta* XP_004994866.1 Excavate

A2 Ep *Exaiptasia pallida* XM_021058503.1 Metazoa

A2 Ce *Caenorhabditis elegans* GI:5923812 Metazoa

A2 Ta *Trichoplax adhaerens* JQ844127.1 Metazoa

A2 Sp Strongylocentrotus purpuratus XP_783963.3 Metazoa

A2 Dm *Drosophila melanogaster* NP_730906.1 Metazoa

A2 Hs *Homo sapiens* GI:13489073 Metazoa

A2 Mm *Mus musculus* NP_444437.2 Metazoa

A2 Cj *Coturnix japonica* XP_015713334.1 Metazoa

A2 Xt *Xenopus tropicalis* NP_001015960.1 Metazoa

A/B Am *Alexandrium minutum* GW809377.1 Alveolata

B3 EsB *Ectocarpus siliculosis* CBJ27890.1 Stramenopile

B3 Sp *Saprolegnia parasitica* SPRG_14244 Stramenopile

B3 PuB *Pythium ultimum* PYU1_G010659 Stramenopile

B3 Pn *Phytophthora nicotianae* KUF77860.1 Stramenopile

B TeA *Trichodesmium erythraeum* 4244466 Cyanobacteria

B TeB *Trichodesmium erythraeum* 4242859 Cyanobacteria

B2 Pm *Perkinsus marinus* XP_002773980.1-3982.1 Alveolata

B2 VbB *Vitrella brassicaformis* GI:873236255 Alveolata

B2 CvB *Chromera velia* Cvel_13030 Alveolata

B2 SmB *Symbiodinium microadriaticum* OLP96595.1 Alveolata.

B1 Bbe *Besnoitia besnoiti* PFH37084 Apicomplexa

B1 NcB *Neospora caninum* GI:820693727 Apicomplexa

B1 TgB *Toxoplasma gondii* TGME49_214620 Apicomplexa

B1 HhB *Hammondia hammondi* GI:675129670 Apicomplexa

B1 Cs *Cystoisospora suis* PHJ23109 Apicomplexa

B1 SnB *Sarcocystis neurona* SN3_00600535 Apicomplexa

B1 whale *Physeter catodon* XP_023973732.1 (Apicomplexa)

B1 Ef *Eimeria falciformis* EfaB_PLUS_53301.g2771 Apicomplexa

B1 Cc *Cyclospora cayetanensis* OEH77492 Apicomplexa

B1 Et *Eimeria tenella* NODE_2204_^1^ Apicomplexa

B4 Ta *Theileria aqui* XP_004830141.1 Apicomplexa

B4 Bbi *Babesia bigemina* CDR97790 Apicomplexa

B4 Pr *Plasmodium reichenoi* GI:832040889 Apicomplexa

B4 Pb *Plasmodium berghei* XP_676226.1 Apicomplexa

B4 Ca *Cryptosporidium andersoni* OII77012.1 Apicomplexa

B4 Cp *Cryptosporidium parvum* XM_627176 Apicomplexa

B4 Gn *Gregarina niphandrodes* XP_011128945 Apicomplexa

B4 Sc *Stentor coeruleus* OMJ70290.1 Alveolata

B4 SlB *Stylonychia lemnae* CDW75041 Alveolata

B4 Pt *Paramecium tetraurelia* XP_001446029.1 Alveolata

B4 Ppe *Pseudocohnilembus persalinus* KRX11086.1 Alveolata

B4 Tt *Tetrahymena thermophila* TTHERM_01014560 Alveolata

B4 Im *Ichthyophthirius multifiliis* GI:471221745 Alveolata

^1^ NODE_2204_length_1392_cov_13.844110; NODE_10691_length_2077_cov_9.128069; NODE_2896_length_969_cov_15.135191

Sequence databases sources:

NCBI

DOE JGI

ToxoDB

CryptoDB

Broad Institute of Harvard and MIT

Lg 1 MALRDV**LI**DG**L**c-----------------------------------------------------------------

Pp 1 MEsKt**LL**nt**L**s-PqLqALVEA--------sLGAK------------------------------------------

PuA 1 MDKKS**LL**SV**L**S-PQLQALVEA-------------------------------------------------------

Cr 1 MNFSVNTAEGGDVEVVVC**I**HAPCPAPDVEITGDDCNVLVVTAEGLTTPLSVPLPTGVDPDTARVRWRG-----------------

SlA 1 MIDPRDVIQ**I**DS**L**D-----------------------------------------------------------------

Dd 1 MDISNLPPHIRQQ**IL**G**LI**SKPQQN-NDESSSSN---------------------------------------------------

Rf 1 MKKKtqVLPKAsssFLKKYPWssFPEnVqMsqtKqEELAFELLPPELKLK**IL**EK**L**nLVDGVLsGtVEAPPLMDFsMPtKIGKssqqKKKKEqtIDnEEnnsKEERVEtHstIIttMHPKtt

VbA 1 MQKNVNEAVFFS**LL**QY**L**DTRP**I**RK**L**KPQDLECVFDDILNDDSK----------------------------------------

CvA 1 MHDKTLNHHV**L**VG**L**HRKDVVRKVALEFFTWIVGRNEEYCCKQSRFCDIDLQLAEEGTDKETPSRCMCLQKKSVDGTKLQK

EsA 1 MALPAKVqAs**I**MsW**L**DEsstAIA----------------------------------------------------------

Rg 1 MDPSLLPSHIQLS**IL**QH**L**GATANR------------------------------------------------------------

Ng 1 MDLTNLTPDLKLK**LL**SM**L**SSLNDADDS---------------------------------------------------------

Mc 1 MDAVSSALKSLSTMPASVQ**L**KV**L**SHMT--------------------------------------------------------------

Eh 1 maehvqslpeslqlr**il**sl**l**tesapaas----------------------------------------------------------

Vc 1 MDLTERINLLPDTVRRQ**IL**QAVCGNSSS------------------------------------------------------------

SmA 1 MPRcLRHKqR**L**As**L**As----------------------------------------------------------------

SnA 1 MRGESMEAWLEACS**LL**PD----------------------------------------------------------------

NcA 1 MG**L**DE**L**FMK----------------------------------------------------------------

TgA 1 MA**L**ED**LL**LK----------------------------------------------------------------

HhA 1 MA**L**ED**LL**LK----------------------------------------------------------------

Mb 1 MIHqHRqqEREREsqREKKsHcMqAGMHcRYMLtIDLsDMYWsDIILFLLsRcLsLsLsLsLsLsLsLsLs---------------------------------------------

Sr 1 MKRnsqtqtnKRtAMstRRAqARALVEqAIAAAAAAADsGVGGsGKGGMDtEtKA------------------------------------

TeA 1 MYTEPKVNKNHDFNDHNIVTIERVNKLLFQSIDT**L**EE**IL**K-NQELPTQERA---TVALK----------------------------------------------

TeA 1 MITTVSESEPQEVKIQLLLAGGHQYTIYLKSDAP**LL**Q**LL**A-KTLLSKHQKNENSTLFQI----------------------------------------------

______________**ZF MYND subdomain**____________

Po 1 MAAVYLS**C**AY**C**G-ATSQQRPHPFT**C**SR**C**LD**V**K**YCS**KD**H**QLRH**W**REA**H**RVE**C**R-----------------------------------

Ep 35 ERARFIPqHRMRLYLKLIncPtKPPKGML**c**DL**c**EqVIPERAKGRYK**c**tR**c**K-AR**Ycs**KD**H**LKtAsKF-**H**HRK**c**s-----------------------------------

Ce 1 MssAPnDDcEIDKGtPstAsLFttLMLsqPssstAVLq**c**tY**c**G--ssctssqLqt**c**LF**c**GtVA**Ycs**KE**H**qqL**DW**Lt-**H**KMI**c**K-----------------------------------

Ta 1 MAscnEI-----------PLcEsVtDGHLLVDqDAY**c**sL**c**F-----KKLIsVK**c**sL**c**qsAY**Ycs**KE**H**qqsH**W**KI-**H**REn**c**M-----------------------------------

Sp 1 MDIHGVR-DKsGFGKEAVEAKPEtPssLVssEAVcAYEL**c**s-----VMsKL-s**c**sR**c**KDVR**Ycs**KE**H**LEn**D**LHs-**H**KKV**c**R-----------------------------------

Dm 1 MsRGRGK-------VRDsAsHsGsHsAsKsAMDPPR**c**sI**c**G-----tqqqLLR**c**AK**c**KAVY**Ycs**PA**H**qHLH**W**PD-**H**RtE**c**R-----------------------------------

Hs 1 MAnDsGG----------------PGGPsPsERDRqy**c**EL**c**G-----KMEnLLR**c**sR**c**RssF**yc**cKE**H**qRq**DW**KK-**H**KLV**c**q-----------------------------------

Mm 1 MAsDsGG----------------PGVLsAsERDRqY**c**EL**c**G-----KMEnLLR**c**GR**c**RssF**Yc**cKE**H**qRq**DW**KK-**H**KLV**c**q-----------------------------------

Cj 1 MAnDsGGGAGqGGGGsssGGGGsGGGssssERDRqY**c**EL**c**G-----KMEnLLR**c**GR**c**RssF**Ycs**KE**H**qRq**DW**KK-**H**KLI**c**R-----------------------------------

Xt 1 MAGGG----------------sEGsnqsERDRqY**c**EL**c**G-----KMEDLLR**c**GR**c**RssF**Ycs**KE**H**qRq**DW**KK-**H**KLF**c**K-----------------------------------

Sc 1 Mn**c**AY**c**YK----sDtKLsq**cs**K**c**HRAs**Ycs**qn**c**qKLH**W**tt-**H**KPV**c**n-----DAnLIEWPPALVEEFLELFIEHLDV-----

Tt 1 MK**C**NL**C**Q-----KEGDLSF**CS**K**C**KKVT**YCS**RE**C**QVED**W**KN-**H**KLV**C**GKQNVSVENQKENKLLLVQDDQNVLIKILRKHFI

**unknown**

EsB 1 MEVLcEADAsnPA-qVEKnAEAIKqR**Gn**EH**F**MAKRHKE**A**VEc**Y**s**qA**IsLs--Asn**H**VLYG**nRs**AAHGG**L**GAWAL**A**AA**DA**RRsVE**I**E**P**GYt**KG**FY**R**LAqALIALDEsAEAEsAIEnG--

Sp 1 MVLEAKADGSFDVDAAGDDWEALKAE**GN**GL**F**AQKDYVA**A**IDV**Y**AR**A**LAAAPTDQQ**H**LVLG**NRS**SCFFQ**L**QRYSD**A**LA**DA**NAALA**L**Q**P**QWE**KG**RA**R**KQ---------------------

PuB 18 SDALSSARTSKKQQAEHFRQK**GN**DA**F**RDRAFER**A**ETF**Y**S**QA**VERD--CDN**H**LLYS**NRS**AARHQ**L**KRFAP**A**LE**DA**QKAID**L**A**P**KWA**KG**YL**R**KAHACEGLRQWGSAIDAYKTA--

Pn 1 MADLIVEAGADGsFDA-sAADqAKALRqq**Gn**EA**F**qqRKFqE**A**KDL**Y**t**qA**IELq--nGn**H**LLFG**nRs**AAcHH**L**KEYEE**A**LE**DA**EKAIE**L**s**P**KWA**KG**YL**R**KAAAcEsLqDWnEAIAAYEqL--

**D1 subdomain**______________________

VbB 1 MDGKVE**V**E**WDE**EtVDKMVAVLRst**L**DHRA---ML**DLERR**A**R--sGtGtE**EKG----------------------------------

CvB 11 QKDGANPVAATAQQASHIKPWVKGPSDGVDPSK**V**A**WDE**GTTQTLIRYALFI**L**DRRT---CL**D**M**ERR**I**R**--**SGTGTE**HLQ----------------------------------

Pm 1 MtVAnRVEDVKELVqsRGKLRED**DE**IALKVtVDLIVRAFVEns**R**q-Lq**DIERR**V**R**--**sGt**n**tE**PRA----------------------------------

SmB 302 -------WKEVqcnPDKGFLEKAItKEAtERVKstPsGKKE**WDE**RtLDttVKVLEEI**Ln**ERs**R**RAKn**DLERR**L**R**--**sGtG**VREFR----------------------------------

Bbe 55 -sAPARRDAKDsPPVGsAAKH--AsPqsssKVPVtLGPs**V**L**WD**K**DVEDA**FVA**Ac**LKV**LnY**st**R**K---**DLERR**t**R**--**sGtGtE**DKG----------------------------------

NcB 60 ----PRSKAKRDAPRASAPTAVSSTLPETGKAQHKLGPS**V**V**WD**K**DVEDA**LVA**AC**LRV**LNY**ST**R**K---**DLERR**T**R**--**SGTGTE**EKG----------------------------------

TgB 77 ----LAAGEKREARCASSSGS--TAAARDSRTKCKAGTS**V**V**W**AK**DVEDA**LVA**AC**VKV**LNY**SA**R**K---**DLERR**T**R**--**SGTGTE**EKG----------------------------------

HhB 77? ----PAAGEERDARCASSSGS--TAAARDPGKKSKAGTS**V**V**W**AKE**VEDA**LVA**AC**VKV**LNY**SA**R**K---**DLERR**T**R**--**SGTGTE**EKG----------------------------------

SnB 125 ----PSAAGRERAAGAPVSTQ-----------GGERQKT**V**A**WD**L**DVE**N**A**LLD**AC**ITS**LNY**AACR---**DLERR**V**R**QV**SG**S**GTE**DRA----------------------------------

WHA ? **sG**s**GtE**DRA----------------------------------

Ef 75 --------RSSSKSVSSNCSSSNSSSGNCSSSSSSSSSKRI**W**TPEMQ**D**VMVEV**C**LRT**LNY**GT**R**R---**DLERR**V**R--SGTG**S**E**LKA----------------------------------

Et ? **R--SGTGTE**MRA----------------------------------

Ta 1 MY**V**E**W**PqsDV**D**RFIEFVKstFDDHD**R**I---n**LE**KcV**R--sGtGtE**HLA----------------------------------

Bbi 1 mPKtKDGKtWq**W**tqAEAsEFFsYARGsFDAGD**R**L---A**L**DKKL**R--**--cAA**E**DAV----------------------------------

Pr 1 MDIV**WD**K**D**TV**D**KFYNYLYDV**INY**EKCV---E**LER**NI**R--**IE**TGTE**DKL----------------------------------

Pb 1 ME**V**E**WD**EAILnqFYnYLYEcFD**Y**EKcL---E**L**EnsI**R--**LEM**GtE**LKMHKEcG-----------------------------

Ca 1 msnklfi**w**sksdietlfsiavdlf**n**dktnr--eairkkvg**--**lrm**g**issnpisye------------------------------

Cp 1 MDKLMV**W**SDEETEKIFQLVVKL**LN**GCETRN--KIMERVQ**--**KR**TGT**RCSSYHQD------------------------------

Gn 1 MAtVsEstssLAqAHDV**V**E**W**PEE**V**VnKFWEV**c**VsVMDLRtAK---q**LE**qKL**R--**K**GtGtE**cLI----------------------------------

Po GAGGKKPTLAEQVANLRLAMLAHAEKTRSISEEIAEAGAERNRILQDVSTASEAVEKVAEQKRNAEREVAAAQSELK--------------------------

Mb LGcWqGEWARGARHGRHRKALKKALDGVERALtqPKsstPDtALKRcVELGqqLGcVEE--------------------------------------------

Sr ALYAqLAKFDDtsDEEEAVAVLnqIAAVLAALqqtPqPqP---------------------------------------------------------------

Ep IsqEDKtsLPLPIGsVqtILPLn--------------------------------------------------------------------------------

Ce sLqtsGMVPsnLMPqAAPAVMAPIPPtVsFDDPALttsLLLsLqnnPILnqtIsnFPPtFsItsKtEPEPsIPIqIPqRIsststVPFssEGsAFKPYRntHV

Ta DERRPKIG-----------------------------------------------------------------------------------------------

Sp tAVGEPssKAsnqstMRAsLPsLRIRKRstGDsnPsLRYYEssssssIssMssssLssGsMsRDLqsLGFMsnDsLnsLsDVnDsqFEqEnEqstGPFRRtts

Dm LLtRqKLnssnnnKqqqRqqIqqLqqAVAsAnLEcsGAGAncstAqMMtPAHqAqsWPAEVDnLLnLLGqPGsqEKAAAA-----------------------

Hs GsEGALGH--GVGPHqHsGPAPPAAVPPPRAGA--REPRKAAARRDnAsGDAAKGKVKAK----PPADPAAAAs-----------------------------

Mm GGEAPRAqPAPAqPRVAPPPGGAPGAARAGGAA--RRGDsAAAsRVPGPEDAAqARsGPGPAEPGsEDPPLsRsPGPER------------------------

Cj GGGAAGAAGGAVEPRGAqPPPPRAHGsRsARGAcGRtEqEAAKGAAPGPEPHGEAAPqDDERVAAAGGGEAAAtAPAPAtEAV--------------------

Xt sDstItPAsqntVKnsKKEqFsssAVsIsHsDtsqFKsHVEPAsGVsDEqEEVDG------------------------------------------------

EsB RADPAAnKEMAALKRRIAGsRRRHtqqVGKGAVAAtGGGGGVPKKAAK--sIDLGRAGLYDD**K**E-APDnntqDqqAnEAGsLKALFssLRDsIVAAssnGsLGnGPARHDL**DG**V**F**s**KL**IEPE

Sp -----------------------------------CILKAMAAAKAAKVTTTANALRGLYVE**K**D--PVVPDEDA-------------TKPAQMLWRRLKQSLAS-QHQGCL**DG**I**F**A**KL**SNER

PuB ISLEAAAQPSTDTK----------RAAVRIKQLEAQLQCEQRAPRAVQKGFFTGQTTSIYAE**K**EDVVKQIVDPINGEARLEDPKERSWRFMLRRLKDGCSNANNAQAVLNG**DG**V**F**A**KL**LQEA

Pn LR---------------------VEsDKsstGAEKAADRVKVLKRnAK--EnKAVKKGFLsG**K**A-KPAIYsEKEGVEqDttqRsWKLMLKKLLDGcnKRGInsRGEsVVLD**DG**V**F**A**KL**LqEn

VbB ------------RHHHHHcHDD---------------------------AADEDDAsDDEGH**H**G**H**DHqHHHH-------------------------------

CvB ------------SKCGHSHGQG-----------------------EACHGHHEGDEDGGEHG**H**S**H**SHGHGHSHGHG---------------------------

Pm -----------------------------------------------VVGAsLFAGPLPKcG**H**H**H**HnE-----------------------------------

Bbe -------RHDG**c**cPPVtAsGsAqPcKHLHGssHsAcssARAssssssAAtEGAtAcctGsPP**H**GRcKAKAAsPDAPsqAVEKPAEAREEsAEs--

NcB -------RIDC**C**ADACSKGCDSSSGAQAPANSPS-----SSKTHACQEAHGDGCSDSS----**HSH**KTHT-------VPAAP----ENSSTASAS-

TgB -------HIDCYDDAPRRHEPSSESHSHAAPP-------QANTHAGSCSDKDSSKDSSKESP**HSH**KKASEVSRCYQSPCGCPSHSPAAAGCSSS-

HhB -------RIDC**C**GAAHGGHDPSSESHSHAAPP-------HADTRAGSCSHKGSSKDSSKESP**H**A**H**KTPSESSS-PPRPSDCPS--AAAASSTTS-

SnB -------RQAP**C**CPTPDAANVADPARAAAASSAG----------------------------**HSH**A-HGGSCCNEKKCGSNGCSADSATTTCTHG

WHA -------RqAP**c**cPtPDAAGVAnPPqAAAAssAG----------------------------**HsH**HtHGGsccsEKKcRsnGcsAHGDttEctED

Ef -------SHSC**C**SASQQQQQEQQQQLDSGCCKK-----------------------------**HS**SCAHGSHAAAG--------------------

Et -------SESC**C**SSYEQQQQQQQQQQHCCGG-------------------------------**HSH**SPSSSAAAAAAADAAADAAAPAAAAAAAP-

Ta ------------------------------------------------------------Fn**H**t**H**EEGtEs------------------------

Bbi -------tqqR**c**Ps------------------------------------------DIqKGFKR**H**EGGVDR------------------------

Pr -------NLKN**C**--------------------------------------------------LCYNKEN--------------------------

Gn -------EIntcs-------------------------------------------------**HsH**EssVAGKsIGEKLGGERKsEEccGH-----

Sc -------------------------HKIIYLIDRsAAqKFKEKt------------HGGGcG**H**K**H**H-----------------------------

SlB 739 --------sIFqKDVLsKRssGsKKHKtnILLKnKsAKIHqKDn------qqtqKALLsLFK**H**sYVDVsKIIGHF-----------

Pt -----------------------nAYKLIFsLDqKqADRFKEKntYYYtqqHKDc------H**H**E**H**KDDDRn------------------------

Ppe 1 ----MsDqEILLVKIFRKYFLEKtEKLnLcnGcKMEsDIFKqKnqYYHDLKHKDK---qKnK**H**D**H**s-----------------------------

Tt 1 ----MKqqEnssDKIqKcnqc--NIYRYIYMIDQKEADQFKQRNTYYYNKKHENQTKQCKHK**H**D**H**SASPHTENKS--------------------

Im 262 ----QENVQNIVVESAKYKIG--N--KYIYLIDQKEADLFKDRNTYYYNKKRENKEQKKNHT**H**C**H**-NNINTENQP--------------------

Ce FnsIssEsMssMctsHEAsLEHMssAsLAMFPtsstAqsDIsRLAqVLsLAGDsPAsLALVttsVPstAstAtIPPPAtttssAtssGKsEtItVGKEKIIqtDDP

DIqIIEtEGGsKPtVsRtRKRPtPsns

Sp sGRGLKP

Hs PcRAAAGGqGsAVAAEAEPGKEEPP

__________________­_____**D2 subdomain**_______________________ ________

Am -----NLEVSAARRAVEDGDF**D**VVQAELQRLR-AALPRD-EAEGLADMVK**A**SLQTAKGRSE-LSD**QG---**--FTQPEWV**E**LPAQ

EsB q**F**RR**I**ALsRLP-EDERRq-A**P**ssFqE**LL**RtPL-**Y**AsA**L**E-KAL**P**R**V**Vst**AA**-s**VL**EG**VK**RR-**G**RE**qG**-----DI**MD**s**AtE**EA**L**K

Sp D**F**VQ**L**VYPGISAEDIQRQQL**P**RS**L**RQ**LL**SDAAT**Y**ESE**L**I-ALM**P**K**VEAKA**N-L**VL**A**NVK**AK-**G**AA**QG**-----EI**MD**A**ATE**AV**LR**

PuB E**F**QK**L**VYPGIP**-**KAQLVH-V**P**QN**L**QV**LL**EDPW-**Y**EDE**L**L-ALM**P**K**VEAKA**Q-S**VL**E**NVK**KR-**G**AA**QG**-----EI**MD**P**ATE**AT**LR**

Pn E**F**qq**L**IYPGIP-KEqLVH-A**P**Kn**L**qt**LL**EDPW-**Y**EqE**L**L-ALM**P**K**VEAKAA**-s**VL**A**nVK**KR-**G**AE**qg**-----dv**md**p**ate**rm**l**l

VbB -----qHHHHHHHHtcKKGPD**D**GV**G**q**LLD**EVc-GDDG**L**R-DMVVL**V**M**ER**tA-EIFE**nV**RRKAAqEE**G---**--AV**MD**EE**t**AIGVq

CvB DEGPKERPVFLKMHK**F**RRGED**D**PV**G**EF**ID**SAV-SQEI**L**Q-QILPK**V**T**E**TTG-GIFTKIVSKVKKED**G---**--HVPNEEEQKA**IR**

Pm -------------stsPAGAE**D**L**IG**DK**ID**ELL-sPss**L**H-DMVPtIIKtst-t**VY**nK**V**VnR-AKEE**G---**--EEMPEnVKsYV**R**

SmB -----sKccGLPDEPtPEAEK**D**IAWR**LI**EDYt-RPAA**L**R-DMAGP**V**M**E**W**A**s-EIFG**nVK**RK-**G**EAA**G---**--qIL**D**AEs**E**IE**I**V

Bbe PAAsYRGVPLPPV**Y**R**F**qKDAD**D**H**LG**A**LLD**E**W**t-GEEK**L**R-q**I**AAE**V**t**ERAA**-L**VY**D**nV**RAK-**G**EAE**G---**--LELGVPE**E**Kq**LR**

NcB SPSMCGGFPAPPI**Y**R**F**QKDAE**D**S**LG**AM**LD**E**W**T-KPAK**L**R-E**I**AGS**VAERAA**-T**VY**D**NVK**AR-**G**EAE**G---**--FSLSGEE**E**KQ**LR**

TgB STAVCGEFPHPPV**Y**R**F**QKDGE**D**G**IG**AM**LD**E**W**T-SLEK**L**R-E**I**AGP**VAERAA**-T**VY**D**NVK**AK-**G**EAE**G---**--FELSD**A**E**E**KQ**LR**

HhB STAVYGGFPPPPV**Y**R**F**QKDAE**D**G**IG**AM**LD**E**W**T-SLEK**L**R-E**L**AGP**VAERAA**-T**VY**D**NVK**AK-**G**EAE**G---**--FELSA**A**E**E**KQ**LR**

Cs 1? MELLPqEDRq**LR**

SnB RKHACAS--SVP-**Y**K**F**KKDPD**D**H**LG**E**LLD**S**W**V-APSR**L**R-D**L**LGRI**AERAA**-K**V**FN**NV**IDR-**G**KAD**G----**MDPPTGPE**E**RQ**LR**

WHA RKHAVVc--PVP-**Y**K**F**KKDPD**D**H**LG**E**LLD**s**W**V-sPsR**L**R-D**L**LGRI**AERAA**-K**V**Fn**nV**IDR-**G**KAD**G---**-MDPPtGRE**E**Kq**LR**

Ef KGETADKTAAPPV**Y**S**F**CKDAS**D**E**LG**KR**LD**D**W**T-SSET**L**K-T**I**VGA**V**C**ER**S**A**-R**VY**N**NV**VAK-AIKE**G---**MTFEKGGPDDRH**L**H

Cc 1? M-qGHtA**V**Ls**R**I**A**-VFLcI**V**FcA-WnAD**G---**LtLERGsEEDRH**L**H

Et SAAAAAAAAAAA-**Y**S**F**CRYEA**D**T**IG**KR**LD**DLL-KREN**L**K-T**L**IPSA**AERAA**-R**VY**N**NV**IQK-**G**KAD**G---**LSMEKGS**A**DDLH**L**H

Ta ------------sHR**F**sKsDD**D**F**IG**qY**LD**DLWDsPEK**L**s-L**I**LqDAI**E**s**A**s-Ks**Y**s**n**I**K**nK-**G**LKD**G**-----LKLtDDI**E**Kn**I**I

Bbi ------------FHsHP-AAD**D**I**IG**EcV**D**ELWssDEK**L**A-AVVADAL**E**n**AA**-tt**Y**ER**VK**qK-**G**InE**G**-----LKVtKEVDVq**L**I

Pr CSEECNKINISS-**Y**K**F**KKDED**D**I**LG**E**IID**NEK-EKEN**I**E-CNIGLITQ**R**VF-SI**Y**T**NV**IKK-AKLE**G----**TYNINLE**T**DNF**IR**

Pb scHEGKYDInIss**Y**K**F**KKDDn**D**L**LG**E**IID**sEK-sEEn**I**K-KsLnnIAE**KA**Y-II**Y**n**n**ILtKEsLKn**G**nRnKEnqI**D**IE**t**DYF**LR**

Ca gelnsesrdkvgvvk**f**asskd**d**i**ig**qt**id**svi-cknf**i**iayvl**p**dtte**k**sh-vi**y**q**n**irqkdhvkrki**-**akdignhlf**t**tfs**l**k

Cp SSCNSKIEGFIGIKN**F**SRSKE**D**K**IG**N**LID**KYL-KKNF**I**Y-M**L**L**P**MIIK**KA**F-LA**Y**NSL**K**EICTGKSNNIYKENEL**D**I-S**E**YN**L**M

Gn VKtGEIRntLtP-**Y**cYKKLKD**D**t**LG**EE**L**qRMc-sRRFFIADHMGL**VAE**s**A**s-sLFEKAtE**K**-RqVAs---------tKEKAA**L**q

Sc ------------sIELD-Entts**L**ER**L**VAEVt-sRsY**L**Y-t**L**I**P**sF**L**qqts**-**EI**F**EKI**K**s**K-G**KqD**G**-----LVW**D**tE**tE**KKV**L**

SlB ------DqnKstEFKKK-nnYFHVER**L**KsDsHKsKHGsnnDGccsAVnKIn**-**qs**F**t**n**IsnR**-G**Kqq**G**-----LDF**D**Eq**t**qKE**IL**

Pt ----cc-GsFFAKV**YI**P-EKERs**L**Eq**I**VFEqL-sKnn**I**R-AKL**P**qILDYLq**-**qR**F**nsI**K**Ks**-G**KKDn-----LqW**D**VE**tE**VY**IL**

Ppe qGG-ccGstFHsKI**YI**PInERnq**L**EKVcGEVF-DKHK**L**V-nMI**P**EFLDF**A**G**-**EK**F**FKI**K**EG**-GI**RDn-----LqW**D**LK**tE**qY**LL**

Tt QNGSCC-TTFFAKI**YI**PDKELTH**I**ERAAKETL-AKHK**L**P-YVL**P**ELLEFIS**-**KK**F**YKL**K**ED**-GL**KDK-----LEWNKQ**T**QTY**LI**

Im KNGQCC-TTFFAKI**YI**PKSEQTH**I**EKSAIEAL-DKHK**L**P-Y**L**L**P**ELLDYIS**-**KK**F**FKL**K**ND**-GI**KDN-----LKW**D**KQ**T**QGY**L**T

**__H1 subdomain___**

¶

Lg -------------------------------------------------------------------------------------------RE-----**G**FAVV**D**D**FL**PsLHcHE

Pa 1 MGRYcRHAADRLsRHRGGtLqWtMHInVnHPLLHRIVDELVDq-----**G**WsHqsI**F**MPERLttR

Pp -----------------------------------LPsPAAsEssHDPsKPqstntDAKtqsnAssPIVLDAsHHAAKLnPsELFtFKHPDsP-----**G**FVIKEA**FL**GHREALE

PuA -----------------------------------------------------------------------SLGVPLQANAPAQPLLKHPESP-----**G**FVVK**D**A**FL**GSEHAVT

Cr -----------------------------------------------------------KTGRLTFTASPAAKATATLASCIAAAAAATAVDD-----AALYA**D**FYCNSPPAVP

SlA --------------------------------------------------------------------------PDVNYLNEDKLLSLIQNEV-----DYLVI**D**NPKDILGEINEYDQFLID

Dd ----------------------------------------------------NKNNLINNEKVSNVLIDLTSNLKIENFKIFNKESLNQLEKK-----**G**YLII**D**N**FL**NDLNKINLI

Rf -----------------------------------------------------EPsPLLFDnIVLnALqEnKsttDsnLcFsLq-nAVnLqKH-----**G**YVVI**D**D**FI**DGnsqMIVncVKqIE

VbA ----------------------------------------------------------------LEALLGYPDGEREQASGSFPRATLPIICG-----SHFVG**D**DVCGE-----

CvA HQCPIVFLCSSATAPVINLTLTMDDVSKLSALSILEALTKKRETLFERTVEEETDPLVFETLGRIGEAVSPQSSAVKQVDLLATLLTTTDDEN-----AFLVGECLTKTGNGRLLKG

EsA ---------------------------------------------------------sGqsEPAAAEDDYsYPVItPADLFsLssGGFAAGsP-----**G**VVVK**D**G**F**REqALq

Gt 1 MVRER-----**G**FAVF**D**G**FL**GRDqAEG

Rg --------------------------------------------------------------IPPKKGGQVEEQETSIDPSTNNIDLKSIPLS-----**G**FIVR**D**S**F**FSPTLTTQI

Ng ------------------------------------------------------KKKNQSSKKSSSDNDYYHHEFSQQDKLFILDLINSIQND-----**G**FFVK**D**D**F**QNEELT--

Mc --------------------------------------------------------PDLYNNPDEEVDSTTVTLASSTSAFDMNLALTELKRT-----**G**VYVC**D**HVFGDAEARR

Eh --------------------------------------------------------------------ptadarlegsyrawahglaarlaah-----rvawq**d**dM**L**PTGAASS

Vc -----------------------------------------------PHTTTPPDARPVAVNHAKDLSRHVEALRSKSSLPAHGILPEIRLTA-----**G**VCIV**D**D**FI**PQKHVKEALVCAQQA

SmA -------------LAALLYFLIPRAIAVLFcGcqtVGcLADVqGKRLFLRRVPDGFDEFVtFDtGsVAEELAqRAALALDAEVVqqLstGDFG-----**G**LVcL**D**nI**L**GPRLsDq

SnA ----------------------------------HLKSRIQAEADCRPVLRHWAAAECPPCLKECRRVHATF[i]SSFSFDS-LKDLLQAGKNG-FPQCFIIVEKVFDDAGVSAAA

NcA -------------------------------LPRHLVSQIKEEADGRP-IVNCTDCSNSDGLERKRRMYPDL---SSFLCDS--SEVDFLVDSANSSAPFVIV**D**KVTEDVGWCRR

TgA -------------------------------LPRHLVSQIKEEADDRP-IFNSADSSTAGGLERNRRVYSDL---SSFLSDS---QFDRLVDPTNKAAAFVVV**D**KA**I**EDADVCRR

HhA -------------------------------LPRHLVSQIKEEADDRP-IFNSADSSTAGGLQRNRRVYSDL---SSFLSDS---QFDRLVDPTNKAAPFVVV**D**KA**I**EDADLCRR

Po -------------------------------------------AKSREIEMCFSMMGGHYDSGYEASSEWGEPPTLTAGQAAS--LAEGLAGR-----**G**WAFI**D**G­­Y**I**DMAVEKMRTC

Mb ---------------MAAALtEIAqAFqGtqPAPVAssAAAAAAAAKqqPHWqHDLALDstnPRYAHVsqLLtEAPDVAEYA-HGLALtLqRq-----HYVVV**D**RV**L**DDHVLRq

Sr ---------------KqRDsDDEDsDDDGDEEKKEEEtAAqGtGsRssGqAqIPREqGqKHsGRYEHVRqLMKDEPEVFAWA-KDLAAqLMRn-----nYVILPnV**L**GPEMAAR

Ep --------------------------------------------------------cGEEAVGLWnHsKt---tnqsLH-IA-KYVVEnLtnY-----**G**HcVL**D**H**F**HGEnIsKt

Ce --------------------------------ADPKInYKDHnKnVVYsttLqEHqKHLqnRGLALsI--HqAMVLRLRYIA-EHVIRsLnEF-----**G**WAVV**D**n**FL**GsDHYKF

Ta ----------------------------------------------------------cDKtGLALEK---tWqLqKnERLs-nMVVnqLntn-----**G**FcIInn**FL**GsscstE

Sp LsELGEtPDtDEnntEEGYMRnVPsVsFILGAPqntGssDsnVItsPtPFsVGVAPqWKIPPntDnGDqsnVPVnVnLsPLV-KYIVKnMMDK-----**G**IcVV**D**K**FL**nDsVGDE

Dm ------------------------------------EtEtGqRqqqHqHHHHnGEKsssYqIGLADAsFMGsGsERRYEDLc-RnIIsDMnqY-----**G**LsVV**D**D**FL**GMEtGLK

Hs -----------------------------------ARssLFqEKAnLyPPsntPGDALsPGGGLRPnGqt---KPLPALKLALEyIVPcMnKH-----**G**IcVV**D**D**FL**GKEtGqq

Mm --------------------------------------------AsLcPAGGGPGEALsPGGGLRPnGqt---KPLPALKLALEYIVPcMnKH-----**G**IcVV**D**D**FL**GREtGqq

Cj -----------------------------------------sLYRDKsnLYPGGVqAGPGGAALRPnGqt---KsLVPqRLALEYIVPcMnKH-----**G**IcVV**D**D**FL**GKELGGL

Xt ----------------------------------GAALKAInEEDDtqVsGEAMGsstsRKVttsGGRPnGqtKP-PLHRIALEYtIPcMnKH-----**G**IcVL**D**D**FL**GqEtGDR

TeA ----------------------------------IIEMTGLLSQKNNYSPATLLSEAQQKLNELQINSLGRNGFNQVPEKIK--FKPLFLSAD------YVEIKN**FL**LPENNQT

TeB ------------------------------------PLAEGHSALCFPSEHLVGMVTEPPISLQ-NLQPQDTKNNLKNSTNT--TPKINNLES-----HYLLIEN**FL**SQSENKQ

_______________________________________**D2 subdomain**____________________________ **H1 subdomain**

Am ---------SCEARTLQRLLLCHVKKRLQQELS**L**APVP**EGG**VALTT---------KYTFAT-----PSSLRHP**G**AATL**L**GEVQ---LRE**L**RRE------**G**LT**V**I**D**KAFD-QASMDR

EsB ---------**PqVLqEAF**A**RE**V**I**GVItM**V**qAtARAqAVIE**AG**KV**A**s**P**-----------Vc**E**E----------**A**s**WD**q**L**HDtt-----VAD**L**sRRGR----RFs**VqD**s**FLG**-**EE**WVsP

Sp ---------**PQV**FN**EAF**A**RELL**SMIQR**V**NTQKHALLAQD**A**RFL**ANP**-----------DA**E**C-------AHL**A**RI**D**DVVGDR-----**L**FRSCSV------PCG**V**L**D**G**F**M**G**-D**E**YIPF

PuB ---------**PQVLQEAF**G**RE**V**L**AMVHR**V**NTKKHALLAND**A**RTL**A**D**P**-----------QS**E**L----------**A**M**WD**Q**L**TS**E**CLDDLLRSDRNDPDSGKVA**G**A**AV**V**D**A**F**M**G**-D**E**WTQL

Pn ---------**pqvlqeaf**G**RE**V**L**AMVRR**V**nYqKHVKLAnD**A**R**L**V**A**D**P**-----------nsDF----------**A**t**WD**q**L**DD**E**FLDELF**I**EKsDVR------**G**V**AV**M**D**E**F**M**G**-**EE**WtqL

VbB ---------KEI**LKEA**L**IR**q**IL**qtVnAK**IR**sAqsLE**sREGGLLA**t**P**----------GGY**R**G----------sD**LD**R**L**Rs**Et**-----IHs**LM**Rt------**G**F**AVqD**G**F**M**G**Gq**E**YtE

CvB ---------AEIF**KEA**LV**REI**QAWVAAQV**R**LSTSGGAV**EGGLLA**T**P**----------GGYST----------**GPL**NN**L**QN**ET**-----VAS**LM**QK------**G**W**AVQD**GW**LG**-GDLHER

Pm ---------REc**L**MnsAKq**EI**VARARKW**L**qGRnqss**s**t**EGG**tY**AnP**----------LALKR-sPVcEVDGtHP**LD**R**L**ns**At**-----IEs**LM**Rn------**G**W**AVqD**G**FI**S-M**K**EVGK

SmB ---------RD**V**V**KE**cL**IR**H**IL**KVInt**E**nsEAHAsG**s**HDA**GLLA**t**P**----------qGYtA----------**G**D**LD**s**L**sVDc-----VnG**LM**tK------**G**YGI**qD**n**F**VD-DRVVED

Bbe ---------qD**VLKEA**LV**REI**HARIns**ELR**R**ERL**R**D**c**REGGLLAnP**----------nGYRD----------**GPLs**Y**L**AnD**t**-----IRD**LM**KE------**G**V**AVq**RG**FLG**-**E**AM**R**KK

NcB ---------QD**VLKEA**LV**REI**HACICT**ELR**R**ERL**R**D**C**REGGLLANP**----------KGYRE----------**GPLS**Y**L**TN**ET**-----VRN**LM**KE------**G**F**AVQ**HG**FLG**-**EK**M**R**QK

TgB ---------HD**VLKEA**LI**REI**YSRICT**ELR**R**ERL**R**DCREGGLLANP**----------KGYRE----------**GPLS**F**L**TN**ET**-----VHD**LM**KK------**G**F**AVQ**RG**FLG**-**EK**M**R**QK

HhB ---------HD**VLKEA**LI**REI**YARICT**ELR**R**ERL**R**D**C**REGGLLANP**----------KGYRE----------**GPLS**F**L**TN**ET**-----VHD**LM**KK------**G**F**AVQ**RG**FLG**-**EK**M**R**QK

Cs ---------LD**VLKEA**LV**RE**VqsRVLKq**LR**q**ERI**L**DsHEGGL**V**AnP**----------sAYRE----------**GPLs**Y**L**tn**E**A-----IRD**LM**Kq------**G**V**AVq**HG**FLG**-**EE**M**R**KK

SnB ---------LEI**LKEA**TI**RE**VQLRVVE**E**VHE**ER**KR**DSREGG**MF**ANP**----------LAFKQ----------**G**H**LS**H**L**LPD**T**-----VAA**LM**KE------**G**V**AVQ**HA**F**V**G**-**EK**V**R**KM

WHA ---------LEI**LKEA**tI**RE**VqLRIVE**E**VHE**ER**KR**DsREGG**M**LAnP**----------LAFKq----------**G**H**Ls**H**L**LPD**t**-----IAA**LM**KE------**G**V**AVq**HA**F**M**G**-**EK**V**R**KM

Ef ---------DEIF**KEA**LL**REL**QLRIAH**ELR**KD**RL**Q**DSREGGLLANP**----------SGYQE----------**GPLS**Y**L**SP**ET**-----IRG**LM**VE------**G**V**AVQ**HG**FLG**-**EE**M**R**KN

Cc ---------EEI**LKEA**LL**RE**tqFRVnq**E**M**R**q**E**q**L**L**DsREGGLLAnP**cRLMqsAcAKKcWKnDVVcAAAYDE**GPLs**Y**L**Rs**Et**-----IRD**LM**VE------**G**V**AVq**HG**F**V**G**-**E**AM**R**Rq

Et ---------EEI**LKEA**LV**REI**QLKVAH**E**M**R**Q**E**Q**L**L**DSREGGLLANP**----------SAYDE----------**GPL**CF**L**KP**ET**-----IRD**LM**VD------**G**V**AVQ**QG**FIG**-**EE**M**R**RL

Ta ---------EtsW**K**RsLnns**L**RKsLVGK**L**qE**E**c**L**qI**s**nD**G**HKF**A**LG-------------------------sEH**n**n**L**EG**E**F-----VYn**LM**nL------**G**F**A**HcsP**FIG**-Kntn--

Bbi ---------qtAW**K**q**AF**tMs**L**sKFAMLD**I**qKWcAsRAAD**GGL**c**A**Ls------------------------EEDYMDFtGn**t**-----IHs**I**VnE------**G**LGIVAGYAA-VDRsA-

Pr ---------RDIFRLV**F**HKY**IL**QNTRNK**I**KQIQCK**D**TKNIIS**LANP**----------LHIKD----------VE**LN**K**I**NSD**T**-----IAN**LM**NN------YIGI**Q**KN**FLG**-KQYMNL

Pb ---------tYI**L**KtV**F**nKY**II**tnVKKRIKEIHcKstKDIIsL**AnP**----------LnIKE----------HD**Ln**K**I**qs**Et**-----**I**Gn**L**MnF------nVsI**q**LD**F**M**G**--KYMKL

Ca ---------laifteslichftnlaidsikkinsqnildis**l**f**a**d**p**----------lcykg---------plk**ln**n**l**vcs**e**-----**l**dky---------**g**islfsgw**lg**-**e**dl**r**sk

Cp ---------IT**V**FSESIIYYCVDKIMD**E**VKNNYSSKTCDISRW**A**S**P**----------YIIKD--------IVNI**I**DNQMLY**E**-----**L**NEF---------**G**ITMKSTWF**G**-**EE**M**R**NK

Gn ---------GFc**LK**L**A**IEqV**I**VqKVKKLRtRsEqMtVKD**GG**R**LA**Y**P**----------qsYKI------MFPKsHYRE**L**nsVPADn--MAE**LM**DR------**G**WtIVtnWMn-c**E**DHPL

Sc ---------E**q**VFD**EA**IsRR**L**VAEVnAKY**R**K**E**EEsF**s**Kq**P**M**LLA**Y**P**----------sGYKE-------DtntE**Ln**I**L**GIDI-----**I**qG**LM**En------DM**A**YIDD**F**FD-YDtAHG

SlB ---------sKcMY**E**sVnKI**I**YEHVKKn**L**Vq**E**EqcY**s**Kq**PGL**F**A**H**P**----------sAIKAM-------KD**G**K**Ln**q**L**DEqF-----**I**KD**L**qsn------**G**YGIKqD**FI**YEKGLIEK

Pt ---------nE**VL**R**E**GLqFYF**L**KEIRL**E**MqK**E**qYI**Ds**KqqM**L**V**A**t**P**----------qtWIn------KYPKIE**Ln**F**L**sADF-----MYD**L**Mnt------**G**Ws**V**MnK**FI**nnnDYcHA

Ppe ---------HE**V**MKq**A**VEnFF**L**q**K**IKKL**L**qKDqKI**D**nAq**P**L**L**L**A**t**P**----------AGWEq------qnPKIK**Ln**F**L**sPDs-----**L**Kq**L**HED------nYtIIPn**F**tKnLDYVRA

Tt ---------GEC**L**KGGCQQFF**I**N**K**IKEYSLKDQRI**DS**KQ**P**L**LLA**TA----------TTWKA------NNPKIP**L**EY**L**SK**E**S-----**L**SEFMTK------**G**YTYIKKYVNADKFVNN

Im ---------AEC**L**KGGCQQFF**I**Q**K**IKEFSIRDQSIET------C**N**S----------FDLEK------NHPKTQ**LN**F**L**QSQN-----**I**SQ**L**INQ------**G**YTYIKN**FI**NNTDFNIN

Sn insert[i]: DDLLNASSSSPSSAASLASSQSTSAFSASSSSSASSTSPPPSPCPSPSSSVAAA

Gn VsRPKRqLRKDKAPAMEtVPIHDL

Cr TDPRPLHGVSPPAASATAAMAELLGLRKAPSDAIRAARKELEVMHEH-

Rf sMKqnGVLKKAGLnRREKHIADVHIsDcGVtKLnGtYHWDAHsKHYVHstsAsctLAPnsDPELsHLFGsKtLtsLsssHtttKP**I**DWV**L**scqHVVFYYGAsVGGEERsVsKDLEPVtsHWqcVAGIEPLPKI

___________________________________________________**H1 sub domain**____________________________ _____

§ ‡ ‡ ‡ §

¶¶ ¶¶¶¶¶ ¶¶¶¶ ¶¶ ¶¶ ¶¶¶¶¶¶ ¶¶¶ ¶¶¶¶¶¶¶¶ ¶¶¶¶

Lg **I**HnA**V**qt**L**EARHVLR**EA**AV**G**RGDRqsV----RtDI**R**R**D**YnA**WL**-----------------------DEDnAPDA**L**MPWFsAMRA--**L**KDA**L**nRELY-**L**GLVE----------------------

Pa **L**AE**E**cRtRAVA**G**DLtP**A**A**IG**RGDGqVI----REG**IRGD**Ltq**WL**------------------------EPGEsEAcDEYLGVMDs--**L**RqA**L**nAsLF-**L**GLED----------------------

Pp VRDALtE**L**Ats**G**tFHD**A**KV**G**AGqnLRn----DRAV**RGD**R**I**H**WI**---------------qtPsDLnAPAqsIHPA**I**LH**L**RRqVEs--**L**VYG**L**RKAsPEMDLRnI---------------------

PuA VRDALLA**L**TKTESF**HEA**KV**G**HGDHLRN**----**ERAV**RGD**R**I**H**W**V---------------KRPSDLNRS-DLLHPA**I**LY**L**MKQVES--AAYAFKQTNPD**L**DLRNV---------------------

Cr YSPG---**L**IWV**G**--K**EA**EA**G**AQISV------NA-V**RGD**VVL**WL**--------DDGALGATAFVKDGVRRPCGFLQ**L**QQ**L**LADVDELVFE-G**L**RPRLAY**L**AGLHRR--------------------

SlA CRS**E**AEQ**L**SQS**G**MLKDSGY**G**KDSDYVK----DKKV**RGD**QFI**WL**----------SYLAKNFSDQNDQENSQKLQN**L**QK**L**VQQMTS--**L**SAK**I**NMYEKP**I**NQISD---------------------

Dd YDESYNQFKENKLIE-**A**GMNKGTDKWK----DKSI**RGD**Y**I**Q**WI**-----------HRDSNSRIQD-KDLSSTIRN**I**NY**L**LDKLDL--**I**KNEFDNVIPNFNSIK----------------------

Rf ttHsnKKnDEKVqFEnVtDsnsVWR------sEDV**R**s**D**LHA**W**M---------------------HsDDAnIAKD**L**RnVItqIDK--**I**RVW**L**nKHVq-FGcqD----------------------

VbA ARK**E**AEG**L**HEG**G**HFKS**A**GM**G**RPSERRE----DSGA**RGD**E**L**M**WL**-------------------NEASLDLFDLRG**L**KQ**I**AHKIES--VRRR**L**NSVHMRRRPANVPERASADNRPLDEEDGGPFSK

CvA **L**QK**E**IAA**L**EET**G**AFQ**EA**AM**G**T--DRRK----DTNA**RGD**RRI**W**A---------------------RRNEVSDHEA**L**SR**I**VVLLED--FRKSVQKQMEA**L**GSACPAYSRRKHPSLEH---------

EsA AHAAALEVEnR**G**EMKP**A**GM**G**RRDGVWH----Gqqs**RGD**s**I**M**WI**------------------tEGIRGKGELPEG**L**En**L**LMRLsA--**L**RGP**L**nDGcG-**L**PttssAPGLGLVKDt-----------

Gt ARGAAqn**I**FRA**G**LMRR**A**GM**G**KDstAWt----DVRA**RGD**E**I**I**W**M------ssLLERKLsGDLsDEALtRsIscMEVcA**L**LHDFRP-s**L**sLqAVsDsI-VsARDsLVtALsqtFtcEK--------

Rg DALLSQSDRSVLGLRP**A**KV**G**TGAMKRR----DDHV**RGD**E**I**AFV----------------DAAFSEGSAGAGNVT**L**ENVVKLAAK-A**L**QPVVDQINYAFGLTGDAKLRSN---------------

Ng SNTKDQL**L**NMKNELNQ**A**GI**G**EGSAKYV----NTEL**RGD**LFK**WL**---------------------SDSDVPDKTP**L**KETLSKLNN--FRRV**L**NYLFD-**L**ELDR----------------------

Mc **I**AS**E**MRE**L**E----LQPGKM**G**LHWQ-------EESM**RGD**KYV**WL**---------------------KRENTQEESA**L**RR**L**LERMEE--WRVQFNKEAG-FGLTH----------------------

Eh **L**LGAAEE**L**PADA-LAP**A**GM**G**ASVARWR----DESV**R**S**D**R**I**G**W**V---PLEPSSGSGDSPFGALHATSGWSELRAA**L**SDVVAALN---HEAAVRPGGER**L**RLP-----------------------

Vc **L**SEGAQQAAIG**G**ALPL**A**SA**G**RQV--------DTAS**RGD**IMR**WL**-------------------RPCEEVSSGRGP**L**AA**L**LERMEA--**L**RSQ**L**ERQGYSVGGR-----------------------

SmA **L**Hq**E**AKV**L**R--KILqPstYWnR---------WqEG**R**E**D**sYRL**I**---------------DRsscELHGFRGMAtG**I**DL**L**LAVcsR--**L**AEIcPVDGKRVGPt-----------------------

SnA AKE-AQA**L**HSA**G**LFKA**A**GFNTKEGHRTV---DAES**R**G**D**T**I**L**WL**------------------HAKE**L**DASPSLRRAASVFGELLLR-**L**NET**L**RR----**L**EQNVQLEA------------------

NcA ARE**E**AQC**L**RTA**G**RFKQ**A**SF**G**GGSSKCA----DVRT**R**S**D**D**I**V**WI**------------------RRED**L**VALPACSQ**I**VS**L**FEEIRAS-VDKSFLR----**L**GTDLEVDK------------------

TgA ARQ**E**AEY**L**RSA**G**RFRQ**A**SF**G**GGSSKSV----DTRT**R**S**D**E**I**V**WI**------------------RQQD**L**SELPACTQ**I**VS**L**FEEIGVA-**I**DKGFAR----**L**GENLEVQQ------------------

HhA ARQ**E**AEC**L**RTA**G**RFRH**A**SF**G**GGSSKSV----DTRT**R**S**D**E**I**V**WI**------------------RQQD**L**SELPACTQ**I**VS**L**FEEIGVA-**I**GKGFTR----**L**GENLEVEQ------------------

Po WLDRY--SNGT**G**D**F**GR**G**RM**GG**GRSGQNIKYIEEGS**RGD**FVR**W**L------------------SPQDDDCPV**G**YKW**L**RAGMDLLVQGLAR---XCRCAA**L**EGVDTVVA-----------X=N or T

Mb FIH**EV**KsA**H**RD**G**nLKP**G**q**L**AGGRAGAntKYVLGHV**RGD**qVG**W**F-nqDPcHDss[i]-DRsqPRcWq**G**s--**G**LRAFtsRMntLVqLIAA-------FVPELqR**I**Es**R------------------**

Sr **L**At**EV**KtA**H**GA**G**RLss**G**M**LG**GGRtGAntKYILsAV**RGD**KVG**W**F-DEDPcHDtt[i]GAGsGVqcWR**G**t--ALKtFsRKMntIVqLIAs-------LVPELKt**L**Es**R------------------**

Ep **I**Ln**EV**Ks**LH**sK**G**s**F**KD**G**q**L**qs---ttGHGtPnRKV**R**E**D**E**I**t**W**L----------------------n**G**E**E**D**Gc**nA**I**AHHMAItDALLqL-----**c**nYF**I**KAnD**I**En**R**------------------

Ce tAK**E**IER**L**YER**G**L**F**sP**G**q**L**ME--AKHKDEFHIKD**IR**s**D**H**I**Y**W**Y-------------------DGYD**G**RAKDAAtVRL**L**IsMIDsVIqH-----FKKR**I-**DHD**I**GG**R**------------------

Ta VLqq**V**Ln**L**Yqs**G**V**F**sn**G**q**L**AR-------nVsVnR**IRGD**K**I**A**W**I----------------------G**G**D**E**R**Gc**EA**I**KY**L**sscVDsLIsR-----**c**n**G**R**LG**nYM**I**tG**R**------------------

Sp **I**LR**EV**Ks**LH**HG**G**DLtD**G**q**L**VY----sqEHtssqE**IRGD**K**I**A**W**R----------------------D**G**t**E**K**Gc**ss**I**GH**L**IsKMDAIIMq-----**c**q**G**q**LG**nLV**I**nG**R**------------------

Dm **I**Ln**EV**RsMYnA**G**A**F**qD**G**qVVtnqtPDAPAVRGDK**IRGD**K**I**K**W**V----------------------G**G**n**E**P**Gc**snVWY**L**tnqIDsVVYRVntMKDn**G**I**LG**nYH**I**RE**R**------------------

Hs **I**GD**EV**RA**LH**Dt**G**K**F**tD**G**q**L**Vs-----qKsDssKD**IRGD**K**I**t**W**I-----------*--*---------E**G**K**E**P**Gc**Et**I**GL**L**MssMDDL*IR*H*-----***c**n**G**K**LG**syK***I***nG***R****------------------*

Mm **I**GD**EV**RA**LH**Dt**G**K**F**tD**G**q**L**Vs-----qKsDssKD**IRGD**q**I**t**W**I----------------------E**G**K**E**P**Gc**Et**I**GL**L**MssMDDLIRH-----**c**s**G**K**LG**nYR**I**nG**R**------------------

Cj VAq**EV**RA**LH**Ht**G**R**F**tD**G**q**L**Vs-----qKsDssKD**IRGD**K**I**t**W**V----------------------E**G**K**E**P**Gc**qt**I**RL**L**MnsMDDLIRH-----**c**n**G**K**LG**nYR**I**nG**R**------------------

Xt **I**Vc**EV**KA**LH**nt**G**R**F**tD**G**q**L**Vs-----qKsDstRD**IRGD**q**I**t**W**V----------------------E**G**K**E**ss**c**KA**I**GK**L**MnKMDDLIRH-----**c**s**G**K**LG**nFR**I**nG**R**------------------

Am **L**RA**E**CEW**L**DNTMGMLNKDDED-------TCNPLQRKF**D**VPLGS------------------PEIVEACRAETPT**L**FAVLECLRG--**LPVLLEE**ALG----**L**QLRVP------------------

EsB **L**LE**D**sA**R**FqAA**G**K**L**H**P**LG**L**E--------------PEHGE**M**A**W**V--------------------EPDnLEFGF**PAL**HE**L**VVn**LH**A--**L**AF**ELn**L**K**D**P**-----K**L**G**L**R------------------

Sp **L**RS**D**AL**RL**RKQ**G**K**L**L**P**VGPH------------------A**M**RFW-------------------TTESSLNEDF**PAL**ADVVEK**LH**M--**LP**FA**LN**RQFD----**L**R**L**CDETK----------------

PuB **L**LD**DV**Q**R**MARNDL**L**MDVAGDA-------------ALAGS**M**IAT------GASKEEHGGRLRFVEPSECEQEY**PA**VAE**L**LEK**LH**A--**LP**Y**EIN**R**K**R**P**----AQAQ**L**C------------------

Pn **L**Ln**DV**F**R**MAKs**G**s**L**MsAV----------------PnI**D**AKHV**L**sRqHAEnqsEtsPGARIRFVEHqDcsAEY**PAL**AE**L**IEK**LH**A--**LP**Y**EInKK**R**P**----EnAK**L**c------------------

TeA A**I**K**E**AIKRKEQYVESSTTTK-----------ADKY**R**QSYVLFS--------------------------QYF**P**E**L**SG**L**IQSKILEI**L**PEV**L**TQLE--FRPFEISE-------------------

TeB **LL**NY**V**LQRKSDFSPTTTSTK-----------AENY**R**RSLVLYS----------------------------F**P**KFQE**L**IVNRIKEIFPDV**L**NKLS--**I**PVFSIAE-------------------

VbB VYR**E**V**E**M**LE**YAst**Fn**q**V**Yq**q**q----------LsGt**R**s**D**D**M**G**W**F------------------sFA**DIDR**Iq-HKH**LW**GVFEV**L**ss--**LPFELnKKA**n----**L**Y**Lq**Ms**------------------**

CvB **I**YS**E**M**ELLEF**D**G**K**F**A**EV**YQ**Q**Q----------MTGM**R**N**D**RI**CW**V------------------AQSQ**LDRE**D-FS**GL**HV**L**FNK**M**IS--**LPFELNKKA**N----**L**Y**LQ**AN**------------------**

Pm **I**RE**E**I**ELL**E**F**E**G**H**F**Dt**V**YG**q**n----------MVAL**R**n**D**KI**c**FP------------------KYRE**LD**V**E**--tYEWLRWLIsERLqD**LPFELn**A**K**LsGEHKt**L**L**qI**H**------------------**

SmB VFK**E**L**E**M**I**D**F**E**GR**Lsq**V**qq**q**K----------MIGY**R**t**D**KI**cW**V------------------nFEG**LDRE**K-qP**GL**LE**L**F**K**K**M**Is--**IPFELnK**tcs----**L**Y**Lq**As------------------

Bbe **I**Wn**E**I**ELLE**Y**DGRFnEVF**s**q**t----------LH**D**L**R**K**D**Y**McW**M------------------ss**sDLDRE**n-**qqGL**Wq**L**F**K**AMq**A**--**LPFELnKKA**s----**LcLqV**s**------------------**

NcB **I**WK**E**A**ELLE**Y**DGRFNEVF**S**Q**T----------MHEL**R**T**D**Y**MCW**M------------------SS**SDLDRE**T-**QQGL**WQ**L**F**K**AMQ**A**--**LPFELNKKA**T----**LCLQV**S**------------------**

TgB **I**WK**E**T**ELLE**F**DGRFNEVF**S**Q**S----------LH**D**L**R**S**D**Y**MCW**M------------------SA**SDLDRE**T-**QQGL**WQ**L**F**K**AMQ**A**--**LPFELNKKA**S----**LCLQV**S**------------------**

HhB **I**WK**E**T**ELLE**F**DGRFNEVF**S**Q**S----------MH**D**L**R**S**D**Y**MCW**M------------------SS**SDLDRE**T-**QQGL**WQ**L**F**K**AMQ**A**--**LPFELNKKA**S----**LCLQV**S**------------------**

Cs **I**LK**EVELIE**Y**DGRFnEVF**H**q**t----------VH**D**V**R**K**D**Y**McW**M------------------stn**DLDRE**t-**qqGL**Wq**L**FKAMq**A**--**LPFELnKKA**n----**LcLq**Ms**------------------**

SnB **I**LN**E**I**ELLE**Y**DGRFNEVF**Q**Q**S----------LNPV**R**K**D**Y**MCW**V------------------T**LSDLDRE**K-**QQGL**WQ**L**FQSMQ**A**--**LP**Y**ELNKKA**H----**LCLQV**S**------------------**

WHA **I**LD**E**I**ELLE**Y**DGRFnEVF**q**q**s----------FnPV**R**K**D**Y**McW**A------------------t**LsDLDRE**K-**qqGL**Wq**L**FqsMq**A**--**LP**Y**ELnKKA**H----**LcLq**Is**------------------**

Ef VLK**E**M**ELL**DF**DGRFNEVF**Q**Q**S----------ML**D**L**R**K**D**F**MCW**A------------------LM**SDLDRE**K-E**QGL**RH**L**L**K**CLF**A**--**LPFELNKKA**N----**LCLQV**G**------------------**

Cc cLK**E**L**ELL**DF**DGRFnEVF**q**q**s----------VL**D**V**R**K**D**F**McW**M------------------A**LsDLDRE**n-**qqGL**RH**L**L**K**CLF**A**--**LPFELNKKA**N----**LCLQV**G**------------------**

Et CLA**E**L**E**MMDF**DG**K**FNE**L**F**Q**Q**S----------LY**D**I**R**K**D**FI**CW**S------------------S**LSDLDRE**N-H**QGL**RH**L**L**K**CLF**A**--**LPFELNKKA**N----**LCLQV**G------------------

Ta AFs**E**I**EF**M**E**Yn**G**I**Fn**KtDKn-----------nsMYDqcYt**LW**L------------------KssH**LD**KnR-**q**KHMLntLEK**L**Kq--**IPYEL**tH**K**cK----**I**L**Lq**AI**------------------**

Bbi **I**RR**E**LM**F**MqYn**G**L**F**tKnKEV-----------ItPY**R**GcAt**LW**V------------------KAED**L**sP**E**HHKEt**L**K-VIEK**I**KR--V**PFEL**c**K**RcK----**I**M**LqI**I**------------------**

Pr **I**YN**E**LI**FIE**YNNQ**FNE**FNTE-----------YKNI**R**T**D**LF**CW**A------------------YITS**LDRE**K-**Q**KG**L**YK**L**L**K**E**L**SY--**LPYELNKKA**N----**L**S**LQL**S**------------------**

Pb **I**YD**E**LI**FIE**Ynnq**FnE**FnsEY-----------KnI**R**t**D**FY**cW**A------------------YIts**LDRE**K-**qq**G**L**YK**L**LKE**L**sL--**IPYELnKKA**n----**L**s**LqI**c**------------------**

Ca **l**vtqiqky**d**yqal**f**tnyntlt-----------gcykl--kvla----------------------**l**hsakikev**l**ltfy**k**s**l**cs--**ipyeln**m**k**lk----**l**a**l**htl**------------------**

Cp **I**NKALIKM**D**YQ**G**LLTNNSVSK-----------SSFSSNFRYAV----------------------**L**ESDEIQDEMLVYFNA**I**EY--**LP**N**ELN**A**K**LG----RN**L**N**I**L**------------------**

Gn **I**AED**VE**W**LD**MHsL**F**EDqtPFs----------PDPc**R**t**D**FA**LW**I------------------nAAE**I**PAV--LKGMsA**L**sLK**L**ts--**LPFE**M**n**q**K**tq----AK**L**t**I**-**------------------**

Sc tFA**E**AEt**LD**F**DGRF**E**E**IMq**q**K----------IKnI**R**t**D**KM**LW**I----KKDIIDAAVGGKHEsPLH**L**tLsY-c**K**n**L**KK**I**sDVYFs--**LPFELnKK**tK----**L**G**Lq**VI**------------------**

SlB VVsRLtL**LE**IE**G**K**F**qIqKsLL--------PLEqRI**R**n**D**KV**L**PF------------------n**I**nE**I**EKIDEFAq**L**HK**I**tKLVYF--**LPFELn**MHLnY---Mq**LqI**t**------------------**

Pt **L**FK**E**LD**FLE**R**DGRF**E**E**VqtE-------------Lq**R**D**D**KtF**W**L------------------t**L**sqM**D**KDs-FKn**L**YF**I**sHI**L**sA--**LPYELn**s**K**nKD---**L**LV**qI**s**------------------**

Ppe **L**YK**E**LR**FIE**R**DGRF**DFPAGE------------EEL**R**n**D**KI**LW**L------------------D**L**qq**ID**KDq-FsK**L**YF**I**cnI**L**ts--**IPYELnKK**DM----KYYA**L**ss**-----------------**

Tt **L**YK**E**LNY**LE**M**DGRF**D**E**PSIND-----------SKT**R**S**D**RL**LW**L------------------T**L**GE**I**SEKD-FPNVKK**I**AFQ**L**SA--**LPYE**F**N**H**K**TQ----**IC**V**QI**S**------------------**

Im **L**YK**E**FN**FLE**I**DGRF**E**E**QVNE------------KSG**R**S**D**RV**LW**V------------------S**L**SE**I**SDKD-FKNFKE**I**CVR**L**TS--**IPYELN**E**K**TQ----FLA**QI**S------------------

Mb [i] = sGRAELAItEEtAsDPtPttEsAqA

Sr [i] = -qRDELPIADsInDnDsDDEnEGDs

**________________________________________________CD subdomain_________________________________________________________________**

§ § §§ ‡

¶¶¶ ¶ * * ¶¶ ¶ ¶

Lg FEs**q**c**A**V**YP**P-------**GA**F**Y**RR**H**V**D**RFKDs----------------nt**R**q**I**scV**YYLn**P---H**W**EAA-------------F**GG**A**L**q**L**YDREGsP------------------------------

Pa FEcHF**A**L**YP**P-------**GA**Y**Y**qK**H**V**D**RFRD----------------DDA**R**tVsAVL**YLn**D---A**W**LPE-------------H**GG**A**LRL**HLPqR--------------------------------

Pp Vst**q**F**A**IF**PG**D------**GA**RFVK**H**V**D**tYsnV---HKDERGG-IsKDGLV**R**L**It**cV**YYLn**D---q**W**EIE-------------H**GG**Y**LR**VHVKDsKHLPAc--------------------------

PuA **T**ST**Q**F**A**IF**PG**D------**G**SRFVK**H**T**D**TYSTA---HQDESGTSSSSDGLV**R**V**LT**CV**YYLN**Q---D**W**VPE-------------HE**G**Q**LR**VYVKGTSTLLMQ--------------------------

Cr SDAMM**A**I**YPG**K------**GA**RFAK**H**I**D**NTTM-----------------DG**R**R**LT**V**L**T**YLN**P---G**W**KEE-------------Q**GG**A**LRL**FPVRQAGVGVGVGGAAE--------------------

SlA FEV**Q**L**A**SYN**G**S------**G**EY**Y**LR**H**K**D**AFRLD--------ENNLADNQKM**RK**Y**T**L**I**T**YLN**P---NLEQI-------KEVKTNKL**G**Q**LRL**YLKEK--------------------------------

Dd **T**QT**Q**L**A**V**Y**-LN------**G**GR**Y**I**KH**R**D**SFYSS------------ESLTIS**R**R**IT**M**IYY**V**N**K---D**W**KKG-------------**DGG**E**LRL**YTNNPNNTNQKELKQTEE-------------------

Rf **t**qV**q**Vsc**YPG**D------**G**sR**Y**RA**H**L**D**EVYVDDstIqsIRnsEqIsAKKt**R**R**It**A**L**L**YLn**E---R**W**nttE------------c**GG**AV**RI**YLsDGV-------------------------------

VbA SEI**Q**LTR**YPG**G------**G**SR**Y**VR**H**L**D**ASQS-----------------NN**R**L**LT**L**I**V**Y**L**N**P---D**W**QTK-------------H**GG**E**LR**ALTESE--------------------------------

CvA SEV**Q**V**A**IF**P**SY------**GA**RFVR**H**K**D**VSSSK-----------GNSSIHN**R**F**LT**V**I**F**Y**VGD[i]T**W**SPK-------------L**GG**A**LRL**HLRTR--------------------------------

EsA **t**sV**q**L**A**R**YPG**D------**G**RG**Y**VR**H**R**D**tPRsA-----------qDsEEAE**RKIt**A**LYYLn**P---D**W**RPs-------------M**GG**q**LR**VHLDsRPEAAAEEGGPRPAGEEEEKAGR----------

Gt AtF**q**L**A**R**Y**Aq-------**GA**c**Y**VR**H**s**D**VsEtn----------------AG**RRLt**c**IYY**F**n**P---G**W**REE-------------H**GG**V**LRL**YHPKEG-------------------------------

Rg NSM**Q**V**A**R**Y**SD-------**G**TF**Y**KK**H**R**D**SSPMM----------------PG**R**L**IT**V**I**A**Y**FAL---E**W**VDG-------------K**GG**E**LRL**HLPNSHKDAP---------------------------

Ng NSI**Q**V**A**F**Y**TN-------**GA**R**Y**IR**H**A**D**ASPLQ---------------SSD**R**R**LT**F**L**L**Y**F**N**K---EIN----------------**GG**S**L**K**L**YRKERSFDNDIRKPYNYQFKENAEN------------

Mc CKFMA**A**C**YPG**G------**GA**R**Y**VR**H**S**D**VSPLV----------------PD**R**R**IT**A**I**L**YLN**D---G**W**QPA-------------H**GG**S**L**H**I**DTQTGE-------------------------------

Eh EKLML**A**H**YP**R-------**G**GR**Y**VR**H**S**D**VSAAT----------------SH**R**RV**T**V**I**V**YLN**P---E**W**DPS-------------R**GG**Q**LRL**FLPPHSSSGRCSAPCSPPSEGET--------------

Vc PTF**Q**L**A**S**YPG**G------**G**TR**Y**VR**H**C**D**ASLSC----------------PT**R**T**LT**A**I**V**YLN**E--PY**W**EPQV------------**DGG**C**L**T**L**YNVPYTGPLRGCGLAMGEP------------------

SmA PHA**q**L**A**cFAEG------s**A**G**Y**AA**H**t**D**GssMA--ELDVDMPLDqKqMIss**R**RF**t**A**I**L**YLn**E--RE**W**EEs-------------YA**G**AF**R**AFRsKDMsGGDsG-------------------------

SnA NEL**Q**L**A**V**YP**KD------STG**Y**VA**H**K**D**RGASA---------------GRD**R**YV**T**A**I**LF**LN**E---L**W**QPA-------------W**GG**D**LRL**HLRDC--------------------------------

NcA **T**EM**Q**LSV**YP**KG------T**A**G**Y**SA**H**K**D**AGPVS---------------GTD**R**F**LT**A**I**L**YLN**E---D**W**KPA-------------C**GG**C**LRL**YLDDC--------------------------------

TgA **T**EL**Q**LSV**YP**KG------T**A**G**Y**AA**H**K**D**TGSVS---------------GAD**R**F**LT**V**I**L**YLN**E---N**W**KPE-------------C**GG**C**LRL**HLDAC--------------------------------

HhA **T**EL**Q**LSV**YP**EG------T**A**G**Y**AA**H**K**D**TGPVS---------------GAD**R**F**LT**A**I**L**YLN**E---D**W**KPE-------------C**GG**C**LRL**HLDAC--------------------------------

Po QSS**M**V**A**I**YP**AARGGNGV**G**SR**Y**IRRI**DNP**IG-----------------**DGR**IL**T**VMV**YLN**PE--G**W**SLDS------------H**GG**T**LR**VFHAMEDGSEV---------------------------

Mb sEA**M**It**cYPG**n------s**t**R**Y**IR**H**c**DnP**HR-----------------n**GRK**L**t**V**L**F**YLn**E---E**W**qPG-------------**DGG**EV**RL**FVtPDEH-qHAAAPADsDDEtEAsAsPtPsGsVEq--

Sr sKA**M**Vt**cYPG**n------**G**AR**Y**IK**H**c**DnP**HR-----------------n**GRK**L**t**V**LYYLn**D---G**W**KqG-------------**DGG**E**LR**VYAqsKEEAtHDDKPAssRDDGDGDGGGDLDDKV----

Ep sP**AM**V**AcYPG**K------**G**AA**Y**K**RH**I**DnP**nK-----------------**DGR**LI**t**t**IYYLn**q---G**W**DARV------------**DGG**V**L**K**L**FPEqKcFPEsnKt------------------------

Ce sR**AM**L**A**I**YPG**n------**Gt**R**YV**K**H**V**DnP**VK-----------------**DGRc**I**t**t**IYY**c**n**E---n**W**DMAt------------**DGG**t**LRL**YPEtsMt------------------------------

Ta **t**Kc**M**V**AcYPG**s------**G**LG**Y**I**RH**I**DnP**nR-----------------**DGRc**V**t**V**LYYLn**P---n**W**nsqD------------c**GG**q**L**W**L**YPnnEnK------------------------------

Sp **t**K**AM**V**AcYPG**n------n**t**K**YV**K**H**I**DnP**nA-----------------**DGRc**I**t**c**IYYLn**K---D**W**DtKK------------H**GG**R**L**q**I**YPAnqnI------------------------------

Dm **t**R**AM**V**AcYPG**s------**Gt**H**YV**M**H**V**DnP**qK-----------------**DGR**VI**t**A**IYYLn**I---n**W**DARE------------s**GG**I**LRI**RPtPGtt------------------------------

Hs **t**K**AM**V**AcyPG**n------**Gt**G**yVR*H****V***Dn*P***nG-----------------**DGRc**V**t**c**IyyLn**K---D**W**DAKV------------s**GG**I**LRI**FPEGKAq------------------------------

Mm **t**K**AM**V**AcYPG**n------**Gt**G**YVRH**V**DnP**nG-----------------**DGRc**V**t**c**IYYLn**K---D**W**DAKV------------s**GG**I**LRI**FPEGKAq------------------------------

Cj **t**K**AM**V**AcYPG**n------**Gt**G**YVRH**V**DnP**nG-----------------**DGRc**V**t**c**IYYLn**K---D**W**DAKV------------s**GG**I**LRI**FPEGKAq------------------------------

Xt **t**K**AM**V**AcYPG**n------**Gt**G**YVRH**V**DnP**nA-----------------**DGRc**V**t**c**IYYLn**K---q**W**DAKt------------H**GG**L**LRI**FPEGKsq------------------------------

Am ETA**M**LSC**YP**P-------**GA**F**Y**RR**H**L**D**SYEGR---------------DIP**R**KV**T**I**L**L**Y**C**N**R---D**W**KPG-------------D**GG**M**LR**AWLGDK--------------------------------

EsB RA**F**qGs**t**M**L**LR---**L**tE**G**cRVPVR**LD**sVAGG--------------AEt**G**H**KI**sA**VY**FVGRKGGsDADKsqqALsEEGDAInA**GG**q**LRL**KnMEtnAPLAEGIV-----------------------

Sp MEVACS**T**S**L**VC---**L**ET**G**QSQPRR**LD**C**G**PHGDP------------N**D**N**G**Y**KLT**C**VY**CL---GGDEN--------------AL**GG**D**L**EVTPLSSGV------------------------------

PuB AQ**F**AHC**T**C**I**TQ---**L**RQ**G**ERQRLR**LD**C**G**KGD--------------K**D**N**G**F**KIT**C**VY**FF**N**GSGGSNG---------------PRMQ**LR**TDLGAQDDAG----------------------------

Pn Aq**F**VHc**t**A**I**qH---**L**Pt**G**HHqPLR**LD**c**G**tGD--------------K**D**n**G**F**KLt**c**VY**FF**n**AV--DsR---------------tRLK**LR**tsLtE-nAP-----------------------------

TeA IEV**Q**LTAHND-------**G**CY**Y**KI**H**N**D**A**G**SEK---------------TAS**R**E**IT**YV**YY**FYQ---EPKAF-------------S**GG**E**LRIY**DTELKGGGAINHDN----------------------

TeB IES**Q**LTAHNN-------NNF**Y**KI**H**N**D**N**G**SPD---------------TAT**R**V**LT**YV**YY**FYR---EPKAF-------------TE**G**K**L**I**IY**DSKIQGKYYVKAQT----------------------

VbB st**Fq**L**A**c**Y**HAK------**G**tF**YK**R**H**I**D**G**G**YDA-------------Ktn**nGRKItAIYYPn**Pq--**DW**tEs-------------**DGG**A**LRIY**GPRRnPYGRsKGGstPPDGDGsEADDDtPA------

CvB GS**FQ**LGC**YP**AD------**G**SF**YK**R**H**M**D**SSFSP-------------ET**DNGRKITAIYYPN**AR--N**W**KDE-------------N**GG**F**L**Q**I**FPRQRKSQ**Q**EAKKAKGKGKKETKKAAGAEKE------

Pm tqV**q**VGI**YP**ss------KGF**Y**KR**H**M**D**G**G**YGD--------------KDV**GR**tF**tA**VVFM**n**sEG-**D**YVDGG------------**DGG**E**L**A**LY**KEGPtEsAE---------------------------

SmB As**F**HLGcF**P**KD-------**A**Y**Y**KK**H**V**D**G**G**YEt-------------nLn**nGRK**V**tAL**F**Y**A**n**K---s**W**sDs-------------**D**E**G**H**LRIY**RRRPnPF**qL**EKGA--tPFqEDEV-------------

Bbe sHVRLVLMnGt------D**A**EIRL**H**E**D**sL------------------n**nG**VMF**t**I**LY**IAsIDs-nED---------------VHIG**I**EVsGA----------------------------------

NcB **WVFQ**M**A**MFRAD------**GA**Y**Y**KK**H**I**D**A**G**YDP-------------AL**DNGRK**V**TAIYYPN**PP--**DW**QAY--cplssaresek**dGG**F**LRLY**PRRRKDV**QL**REGSAAGPPDAEP--------------

TgB **WVFQ**M**A**MFRAD------**GA**F**Y**KK**H**I**D**A**G**YDP-------------AL**DNGRK**V**TAIYYPN**PP--**DW**EAK-------------**DGG**F**LRIY**PRRRKDI**QL**QEDSAAGPADAEP--------------

HhB **WVFQ**M**A**MFRAD------**GA**F**Y**KK**H**I**D**A**G**YDP-------------AL**DNGRKITAIYYPN**PP--**DW**EAK-------------**DGG**F**LRIY**PRRRKDI**QL**QEGSAAGPADAKP--------------

Cs **WVFq**L**A**V**Y**qGD------**GA**Y**Y**RK**H**M**D**G**G**YEA-------------sV**DnGRKI**s**ALYYPn**At--**DW**qEK-------------**DGG**Y**LR**V**Y**PRRRREq**qL**AEGsAMGADEEEKKEDEEGKDEnVKKR

SnB **WVFQ**L**A**V**YP**GD------**GA**Y**Y**RR**H**M**D**A**G**FSP-------------AL**DNGRKITALYYPN**PP--**DW**KPS-------------**DGG**Y**LR**V**Y**ERWRRKE**QL**GMAPGSVAPYEVATNF-----------

WHA **WVFq**L**A**V**YP**GD------**GA**Y**Y**RR**H**M**D**A**G**FsP-------------AL**DnGRKItALYYPn**PP--**DW**nPs-------------**DGG**Y**LR**V**Y**ERWRRKE**qL**KMAPGDVAPYEVAtsY-----------

Ef **WV**V**Q**L**A**VF**P**GN------**G**S**FY**RK**H**F**D**G**G**FSD-------------FQN**NGRKITAIYYPN**PT--**DW**KEE-------------**DGG**S**L**LV**Y**RRRSRRE**Q**EKDANCLSIKEEEPA-------------

Cc **WV**V**q**MsVF**P**P-------As**FY**RM**H**s**D**G**G**Fsq-------------ERn**nGRKItAIYYPn**AK--**DW**qDA-------------**DGG**V**LR**VFKRRtRKE**q**qtDsscLtPsPDEtPRFVVRLPAcRVLL

Et **W**TV**Q**LSKF**P**QS------**GAF**LKS**H**I**D**G**G**FEE-------------DTN**NGRK**VS**AIY**F**P**CGP--R**W**QES-------------E**GG**S**L**QV**Y**RRRTAAE**Q**QQNSKSVFVAAAEKP-------------

Ta ssLcVV**Y**MKPn------IsRILL**H**n**D**Gs------------------t**nG**V**K**Y**t**c**IY**I**P**KAsG-nDq----------------FIs**L**E**I**cKD----------------------------------

Bbi **WVFq**M**A**MFRAD------**G**AYYKK**H**I**D**A**G**YDP-------------AL**DnGRK**V**tAIYYPn**AP--**DW**KEK-------------**DGG**A**LR**V**Y**PRRRKEq**qL**sEGsAAGsGEAKA--------------

Pr TL**FQ**FL**Y**FSPN------HSFLKN**H**S**D**G**G**YDN--------------L**DNG**K**KIT**C**IY**I**P**SEY--EHN----------------QVL**I**K**LY**KNIN-T**IQ**NNQSRNDTKIKNINDNISSNLENNKP--

Pb tL**Fq**FL**Y**FsPn------KsFLKK**H**AEG**G**YGD--------------M**DnG**q**K**Vtc**LY**I**P**YVH--PDD----------------DVt**I**KVHKsEnGFVsKqKEKnKVDnInqPInVKDsL-------

Ca -pptfhvfsls------qddtlcinkqtia-----------------p**g**s**k**val**ly**f**p**sset-lak----------------itct**ri**ngekgrat-----------------------------

Cp –SKSIYILSIE-----PANTGFIIGKDSES---------------FIP**G**S**KI**GI**IY**FSCKE--KIK----------------INCQNNKTKK---------------------------------

Gn nnnLFVDMYKA------**G**sFFRD**H**A**D**nDVEP-------------ER**DnGR**tF**t**F**IY**ILRsPc-qMR---------------MsALnKtAK-----------------------------------

Sc DsMcLDcFsq-------**G**tFHKP**H**s**D**s**G**FGK--------------D**D**t**G**c**KIt**c**IY**VIs[i]IEIn------------------------ missing exon?---------------------

SlB DH**F**EA**sY**FGGA------HnqHKL**H**A**D**s**G**FGE--------------Fnt**G**L**KIt**V**L**LII**n**DDK-sVD--------------s**D**Y**G**q**L**I**I**EqDGqGnGsEGGEqVq---------------------

Pt EsY**q**I**s**FFGGK------DKKHKK**H**F**D**ssFDK-------------KA**D**t**G**K**K**F**t**F**LY**VV**n**PVtIEIE------------------------ missing exon?---------------------

Ppe EM**Fq**L**sY**FsEH------DtFqKV**H**M**D**sHFDs-------------KK**D**t**GR**t**L**sV**LY**Fs**n**nEF-DLKYs---KtEIEKMqKEKnAV**LRIY**qnEEKsK-----------------------------

Tt EL**FQ**I**SY**FKAN------**G**SFQKP**H**YESSFDG-------------DQ**DNGRKI**NC**LY**FNSLDD-DIDCT-------------NY**G**KV**RLY**T-SVSQN**Q**NEPGD-----------------------

Im EL**FQ**V**SY**FKNN------**G**SYQEK**H**F**D**SSFEG-------------NQ**DNGRKLT**V**LY**FS**N**LDN-DINCK-------------**D**Q**G**K**IRIY**PDFLKDD**Q**NK--------------------------

CvA [i] = PSDVCLGDGNTENEKGGGGEGPVQS

Sc [i] = nssnnt

CvB? KKAEGPGASNEGQKADDAAAPSTVSKDDTKAGEESEEEKE

Cc sLssLcIqLPVRLEAPEqKAIFLssPPcVsAAsRI

EsA? GGssGnAsPLEGnPPqGREEVntstLDGsRntDVnGGGsAPDGLGFAGPRGGKGGEEstVGqGA

**_____________CD subdomain________________________________**

§ §§ ‡ §§ §

¶ ¶ ¶¶¶ * * ¶ * ¶

Lg LAAVL**P**nAn**R**F**V**c**F**---R**s**-D**L**---P**HEVL**Et------------HEK**R**Y--s**Lt**G**W**FKnR*

Pa qV**DI**q**P**t**G**Gs**LV**V**F**---M**s**AGt---E**HEVLP**A------------sRD**R**L--s**Lt**G**W**FRRRnEsLLqLs*

Pp HW**D**VP**P**KL**D**t**LVLF**---R**s**RDV---**EHEV**M**P**t------------YRE**R**K--**A**V**t**I**W**YYGKPsKAssALARtEVLsVPRPLPsItGnEAnHttqPsIFVAIPsYRDsEcRH–gnt1^2^

Pu HW**D**VA**P**KL**D**T**LV**V**F---RS**LDV**---EHEVLP**T**------------**FYE**R**M--**AIT**V**W**YYGHVAKQPPDPAASIE rest is gnt1

# Cr QVDVLPVAGRVAVF---LSAEV---AHEVMPT----------HGAAQRH--AVTLWYFDAGEHAAALAAARVMPGATSKPSAQAGATALLRDLLAEEAASGIPETKDGCAALGTRVA(63)*

SlA IV**DI**V**P**HLN**R**SI**LF**---K**S**ESV---**EHEV**K**P**T-----------VGYQ**RF**--**A**V**T**T**W**FRHIHSASLSKVENKLVSDENSTLFIGIPSYRDPQLVDTIQSIIDNSKYPSK-gnt1

Dd FI**DI**E**P**IA**DRL**L**IF**---L**S**PF**L**---**EHEVL**QCN-----------FEP**R**I--**AIT**T**W**IY*

Rf YR**DI**E**P**VAG**RL**L**LF**---n**s**qW**L**---P**HEV**M**P**V-----------FHRD**RF**--**AIt**L**W**MY*

VbA QV**DI**P**P**LM**D**TF**VLF**---R**SD**M**I**---**EHEVLP**C------------YCE**R**Y--**A**VSC**W**FHVHTDCISSGKDDVA*

CvA QV**DI**A**P**L**G**G**R**F**VIF**---R**SD**LV---**EHEVLP**S------------SHR**R**V--**A**VSS**W**LSFAPERSKTADTGGSAATETLAQSDDEIQQKTADG-GRETKDVQLPVQEEGGERL*

EsA KW**DI**E**P**VL**DRLVLF**---R**sD**LV---**EHEVLP**A------------sAP**R**L--**A**V**t**L**W**FYGRqLGLPAsPstRsPssVPPLPAPEqqLAAGDDtRDKVRPLPsPAGsGAAHAAEA-gnt1

Gt LV**D**VA**P**Lc**DRL**L**LF**---LP-DM---**EHEVLP**c------------AsE**RF**--**AI**sG**W**LYGPssFLEscqAFAARREEEEA-RPEEIFVsIVAYRD-gnt1

Rg FI**D**VK**P**IFN**RL**L**IF**---K**S**-HY---**EHEVLP**T------------FCD**RF**--**A**F**T**M**W**LYSDNPHTIETICKEPPITVPKSITLKEDSTSTIFISIASYRD-gnt1

Ng EATVQ**P**LFN**RL**LS**F**----**S**SEY---Y**HEVLP**S------------LSD**RF**--S**LT**M**W**MYGKKDVWKQINDLTQSTIFISICSYRD-gnt1

Mc RWEVQ**P**LL**DR**M**V**F**F**---T**S**-E**L**---M**HEV**R**P**A------------YKP**R**M--**A**V**T**G**W**YYGPDALQ??

Eh ATVVA**P**LLG**RL**L**LF**---D**S**-T**L**---**EHE**AHHTLAAPSHTTRPTPTQP**R**W--**ALT**G**W**LSVDTAPQRPAPAAPSDTTE—AGSLASILQLAAMATARPASTPTLPTPPPPPAAA-gnt1

Vc ATVVA**P**L**G**G**RLV**V**F**---E**S**-H**L**---S**HEVLP**T------------FRN**R**LWGWLGS**W**LGWLVQRNAWHCTMTVSSLFSQADHPERVRVGVVWQIDANTESSFAAVA-gnt1

SmA FV**D**VW**P**R**G**Gs**LVLF**---RcRD**L**---A**H**q**VL**As------------qHD**R**Y--**ALt**M**W**FtAPAVALFPLsLAAMAWGHRGsMRttGRsRtIFAAFLAAcGIFAHWRcsFVnAWtAR-gnt1

SnA LL**DI**C**P**VANRMI**IF**---R**SD**L**L**---**EH**S**VL**LVL----GVPRPIKENS**RF**--**A**L**TCW**FSVRRSSS*

NcA RL**D**VS**P**RLGK**LV**V**F**---R**S**EQ**L**--**-EH**S**V**M**P**VT-----------CAD**R**Y--**A**I**TCW**FSVRRRAAG*

Tg RV**DI**S**P**CLGK**LVLF**---R**S**EQ**L**---**EH**S**VLP**VA-----------CAD**RF**--**A**V**T**C**W**FSVRPRVAGQSGS*

Hh RV**DI**S**P**CVGK**LVLF**---R**S**EQ**L**---**EH**S**VLP**VA-----------CAD**RF**--**A**V**TCW**FSVRRRAAGRSGIVK*

Po ATG**I**C**P**S**G**G**RL**LV**F**---L**SD**RV---**PHEVLP**VT----------GDLH**R**L--**AIT**T**WY**M**D**FAARARATCEEPSLAETEKLRNEMARMGGHSVDDDVTAA*

Mb AVt**I**P**P**RAntMVI**F**---F**sD**R**R**--V**PHEVLP**t------------nVP**RF**--**A**A**t**H**W**FY**D**ADERAEAEAsA-----GtAF-ELDqqIEEERIRREIEKFEAtq-AqAqMVPAKnqVns(163)*

Sr LV**DI**P**P**RL**D**tMVI**F**---F**sD**K**R**--V**PHEVLP**t------------sKP**R**Y--**A**V**t**H**W**FY**D**LEEKqMAEqqqqqqqGntAnGRsDIAIEEERLRREIERFqAKHGAsAHVLP(179)*

Ep EVH**I**E**P**IF**DRL**ML**F**---**WsD**K**R**--n**PHEV**c**P**A------------YRq**R**Y--**AIt**L**WY**F**D**tnERKLFFEKLKqKqIKHPEsqDsqELDsnDRsRLVKEtE*

Ce PM**DI**D**P**RA**DRL**VF**F**---**WsD**R**R**--n**PHEV**M**P**V------------FRH**RF**--**AIt**I**WY**M**D**KsERDKALAKGKEsDAAcAsKKEnDPtsssLnsLIGsLLRPRKnPstHDLsKLDLR(101)*

Ta VVK**I**D**P**IF**DRL**LL**F**---**WsD**R**R**--n**PHEV**K**P**A------------YAM**R**Y--**AIt**L**WY**F**D**EKERALssqnGt*

Sp VAn**I**D**P**IFn**RL**IF**F**---**WsD**K**R**--n**PHEVLP**A------------RAt**R**Y--**AIt**V**WF**F**D**AVERAKAKsGGMDP*

Dm VA**DI**E**P**KF**DRL**IF**F**---**WsD**I**R**--n**PHEV**q**P**A------------HRt**R**Y--**AIt**V**WY**F**D**AKEREEALIRAKLEnsKtnnLAAqAqAqqAEPDstttPPAAPAssAssLPVsMstGt(20)*

Hs FA**DI**E**P**KF**DRL**LF**F**---**WsD**R**R**--n**PHEV**q**P**A------------yAt**R**y--**AIt**V***Wy****F***D**ADERARAKVKyL-tGEKGVRVELnKPsDsVGKDVF*

Mm FA**DI**E**P**KF**DRL**LF**F**---**WsD**R**R**--n**PHEV**q**P**A------------YAt**R**Y--**AIt**V**WY**F**D**ADERARAKVKYL-tGEKGVRVEL-KP-nsVsKDV*

Cj FA**DI**E**P**KF**DRL**LF**F**---**WsD**R**R**--n**PHEV**q**P**A------------FAt**R**Y--**AIt**V**WY**F**D**ADERARAKVKYL-tGEKGVRVELnKPsDsVGKGVL*

Xt FA**DI**E**P**KF**DRL**LL**F**---**WsD**R**R**--n**PHEV**q**P**A------------FAt**R**Y--**AIt**V**WY**F**D**ADERARAKEKYLntGERGVRIELnKPsEqVVKEVqnP*

Am TVEFE**P**SAG**R**VI**IF**---MAQ**D**I---W**HEV**TES------------RIE**R**Y--**A**V**T**Q**W**VW**D**IKHDSLGR*

EsB VVE**L**E**P**tA**DRLV**M**F**---R**s**DcVstqtL**EVL**GHE------------**R**EqY--AMLF**W**MH**G**ARGGAGDVAGGDGGGGLKtEAEDLsPssqREDGGEEKDVGKGtGL*

Sp PTVVAAKT**DRL**W**LF**---K**S**QEV----LNA**L**TSV---------TSPSPM**F**--IWCFVST**G**WCPCHR*

PuB AQQ**I**T**P**RA**DRLVLF**---Q**S**QRV---FN**E**ITTVP-----------GDEL**F**--FL**T**F**W**IH**G**KELR*

Pn VRq**I**D**P**qP**DRLV**M**F**---R**s**qsV---Ln**E**ItAVH----------EGEDL**F**--YL**t**F**W**VH**G**qRLE*

TeA YKT**I**T**P**VNNS**IV**F**F**---N**S**-RC---R**HEV**M**P**V-----ICPSQAFENS**R**F--TVNG**WI**RRLVS*

TeB FKS**I**E**P**TNNT**IV**F**F**---L**S**-RY---M**HEVLP**V-----TCPSQDFADS**R**F--T**I**NG**WI**RRS*

VbB Iq**DI**L**P**K**GDRLVLF**---**RsR**DM---**PHEVL**Lc------------H**RKRF**--**A**VsL**WL**P**GP**P**GPGDD**Wqt*

CvB EAK**I**S**P**AA**DRLVLF**---**RSR**EM---**PH**A**V**TEC------------H**RKRF**--**A**V**T**L**WL**P**GP**A**GPGDD**LSPPN*

Pm DVR**I**S**P**M**G**G**RLVI**Y---KA**R**DV---**PHE**I**LP**T--------CCSSGGR**R**I--**A**V**T**AF**L**T**G**PQMSDK*

SmB EE**DI**t**P**EAG**RLVLF**---**RsR**DV---**PHEVL**Kt------------KR**KRF**--**A**V**t**L**WL**A**GP**P**GPGD**qPEGHHtsEMLKGtRAtYGLEPRcVEVtAqVtnPEEKVLKKAGVFGKV(110)*

Bbe VR**DI**A**P**A**GD**V**LVL**L---**RsR**DM---**PHEVLP**c------------H**RKRF**--**AI**tL**WM**t**GP**A**GPGDD**V*

NcB LT**DI**S**P**V**GD**T**LVLF**---**RSR**DM---**PH**Q**VLP**C------------H**RKRF**--**AI**TL**WM**T**GP**A**GPGDD**I*

TgB LV**DI**K**P**L**GD**T**LVLF**---**RSR**DM---**PHEVLP**C------------H**RKRF**--**AI**SL**WM**T**GP**A**GPGDD**V*

HhB VA**DI**E**P**L**GD**T**LVLF**---**RSR**DM---**PHEVLP**C------------H**RKRF**--**AI**SL**WM**T**GP**A**GPGDD**V*

Cs tV**DI**K**P**A**GD**t**LVL**L---**RsR**DM---**PHEVLP**t------------Y**RKRF**--**A**VtL**W**It**GP**P**GPGDD**t*

SnB LRE**I**E**P**A**GD**T**LVL**L---**RSR**EM---**PHEVL**ET------------H**RKRF**--**AI**TL**WM**S**GP**P**GPGDD**I*

wha LRE**I**E**P**A**GD**t**LVL**L---**RsR**EM---**PHEVL**Gt------------Y**RKRF**--**AI**sL**WM**s**GP**P**GPGDD**V*

Ef VKRVV**P**K**GD**T**LVL**L---**RSR**DM---**PHEV**TET------------F**RKRF**--**A**VSLY**M**A**GP**P**GPGDD***

Cc IqE**I**K**P**q**GD**A**LVL**L[i]**RsR**EI---**PHEV**sEt------------LK**KRF**--**AI**sFY**M**t**GP**P**GPGDD***

Et IKE**I**K**P**QP**D**T**LVL**L---**RSR**EN---**PYE**IK**P**V------------FK**K**L**F**--LVSFF**M**T**GP**P**GPGD**KCKP*

Ta RHKVPIMD**D**L**LLIF**---**Rs**DI----D**YE**IERV------------sE**K**c**F**--M**I**MA**W**LI**GP**n*

Bbi RK**D**VKLEt**D**M**LI**VL---**Rs**nV----**PYEV**K**P**Vn------------GtV**F**--IVnA**W**Vt**G**tK*

Pr IQV**I**KAE**GD**S**LIL**L---QT**R**NV---S**YE**ISMS------------KE**K**F**F**--MVNL**W**IP**GP**VSVDKHM*

Pb IqI**I**K**P**Ks**D**s**LI**FL---qtRnt---s**YEI**sKtR------------HKF**F**--IVnLsIY**GP**VsLDRnM*

Ca pis**l**evsn**d**k**lii**v---**k**lmen---k**y**t**i**kvng-----------sniiy--fviiyiy**gp**evlk*

Cp SVE**L**ELSD**D**K**L**V**I**A---DLNKN---K**Y**TFRNFE----------SELVTY--CILTYIF**GP**E*

GnA Att**L**nLqPnsM**LIF**---K**sR**RF---K**YEI-P**KE-----------DH**KR**V--M**I**YY**W**cH**G**MnDV*

Sc ntP**I**PLVnn**R**F**II**L---**KsR**Kc---tIs**I**P**P**Vs------------E**K**L**F**--**L**cYYYIL**GP**cDPYq*

SlB qKA**I**nLRnnt**L**VVL---M**sR**KV---R**YEI**KDVK------------s**K**VY--V**I**KGLIs**GP**EDPnKLc*

Pt EqK**I**tLqP**D**n**I**VGL---M**sR**KI---**PY**A**L**qsnD------------GRA**F**--**LIR**YFID**GP**VtnIc*

Ppe HI**DI**EqqA**D**t**L**V**L**L---**KsR**II---**PYEILP**nK-----------tE**K**K**F**--**ILR**F**W**Vn**GP**qDKEnRRF*

Tt FVEYSMEP**D**S**LLIF**---**KSR**LF---**PYEIL**SNN---------SSNS**K**R**F**--VV**R**Y**W**IT**GP**IELNKKF*

Im YVEFETEV**D**S**LLI**L---**KSR**VI---**PYEILP**NN-----------NQ**K**RY--**IIR**F**W**VT**GP**ADKNKKQF*

Cc [i] LHAPRVALLLcERR

^2^ coding region continues to encode gnt1
