# Supplementary figures and images for: A Toxoplasma Prolyl Hydroxylase Mediates Oxygen Stress Responses by Regulating Translation Elongation
Source: mBio. 2019 Mar 26;10(2):e00234-19. doi: 10.1128/mBio.00234-19 (PMC6437050; doi:10.1128/mBio.00234-19)

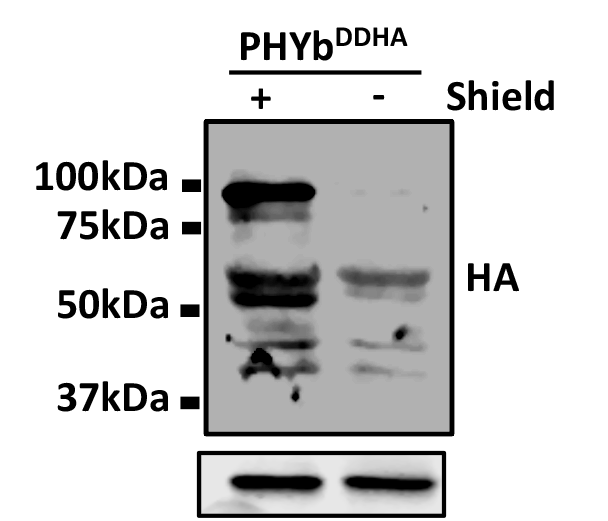

Supplement: FIG S3 [file mBio.00234-19-sf003.tif]

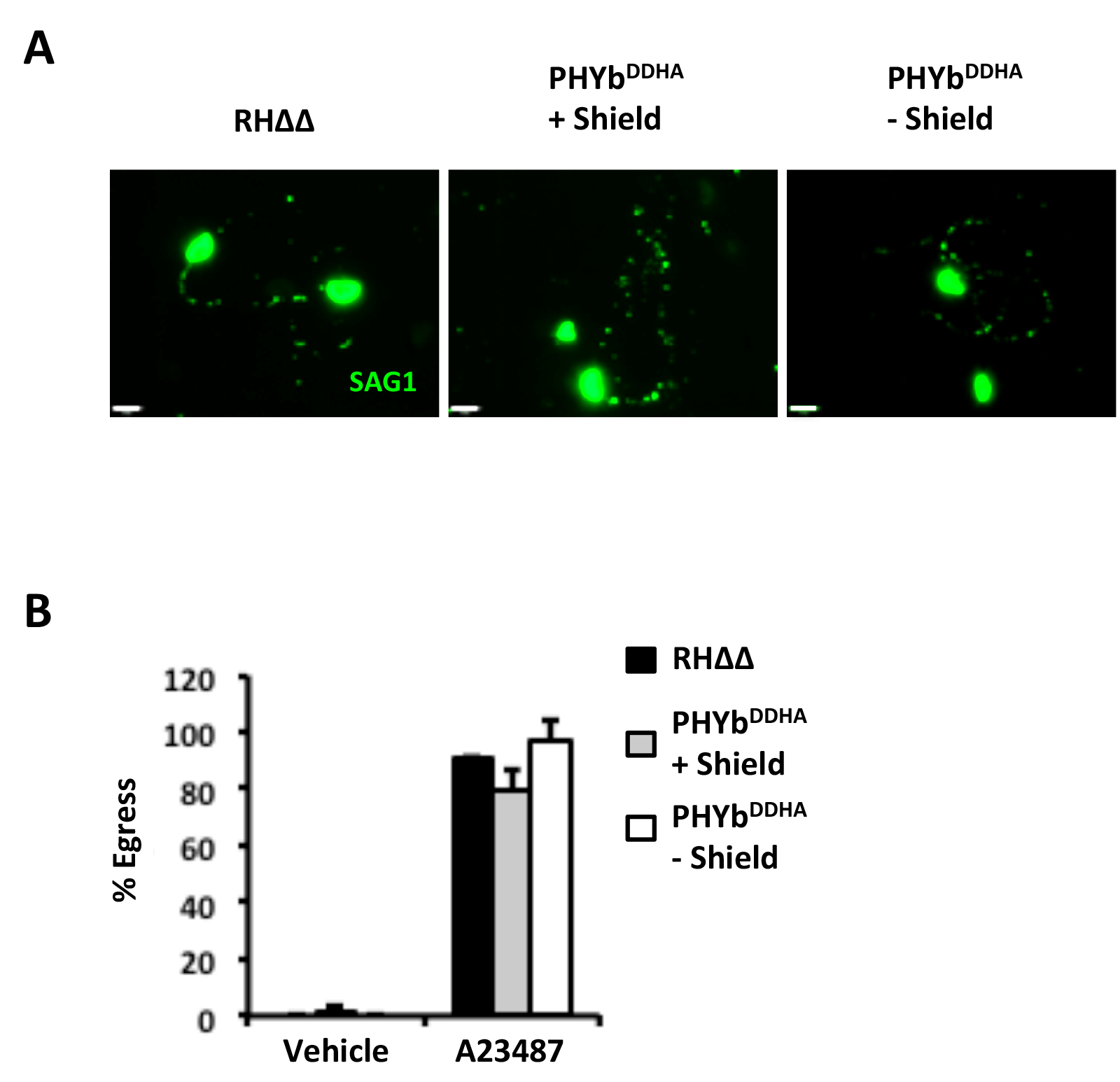

Supplement: FIG S4 [file mBio.00234-19-sf004.tif]

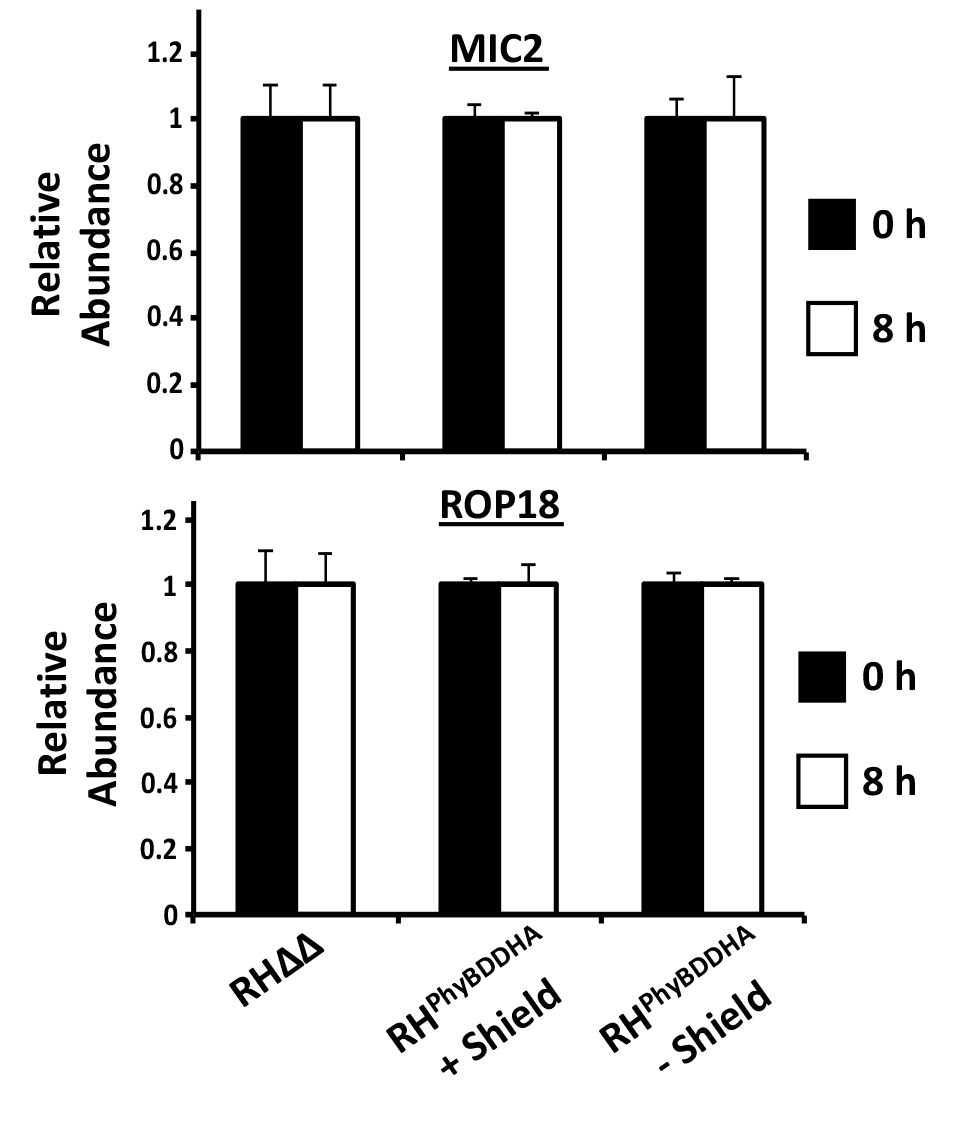

Supplement: FIG S5 [file mBio.00234-19-sf005.tif]

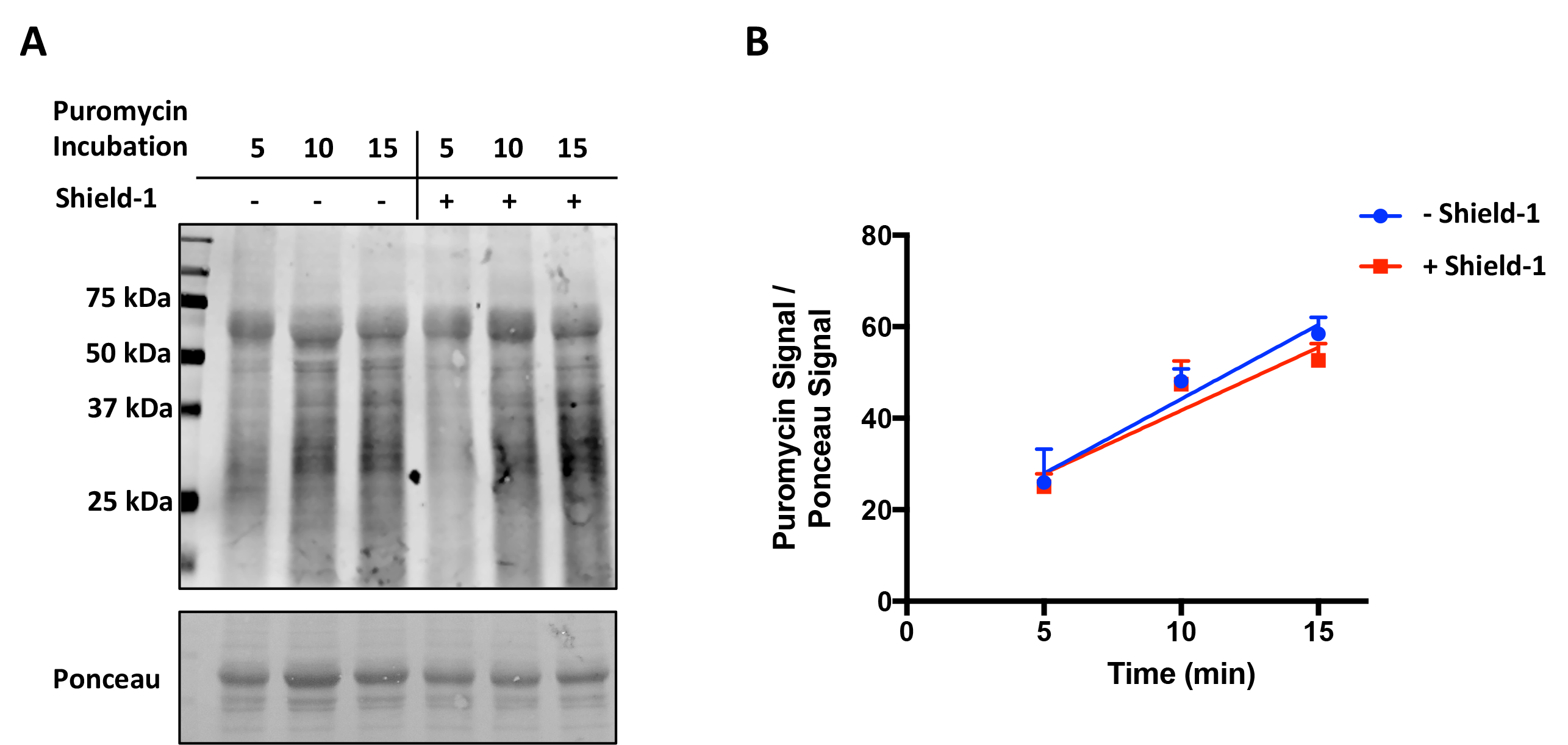

Supplement: FIG S6 [file mBio.00234-19-sf006.tif]

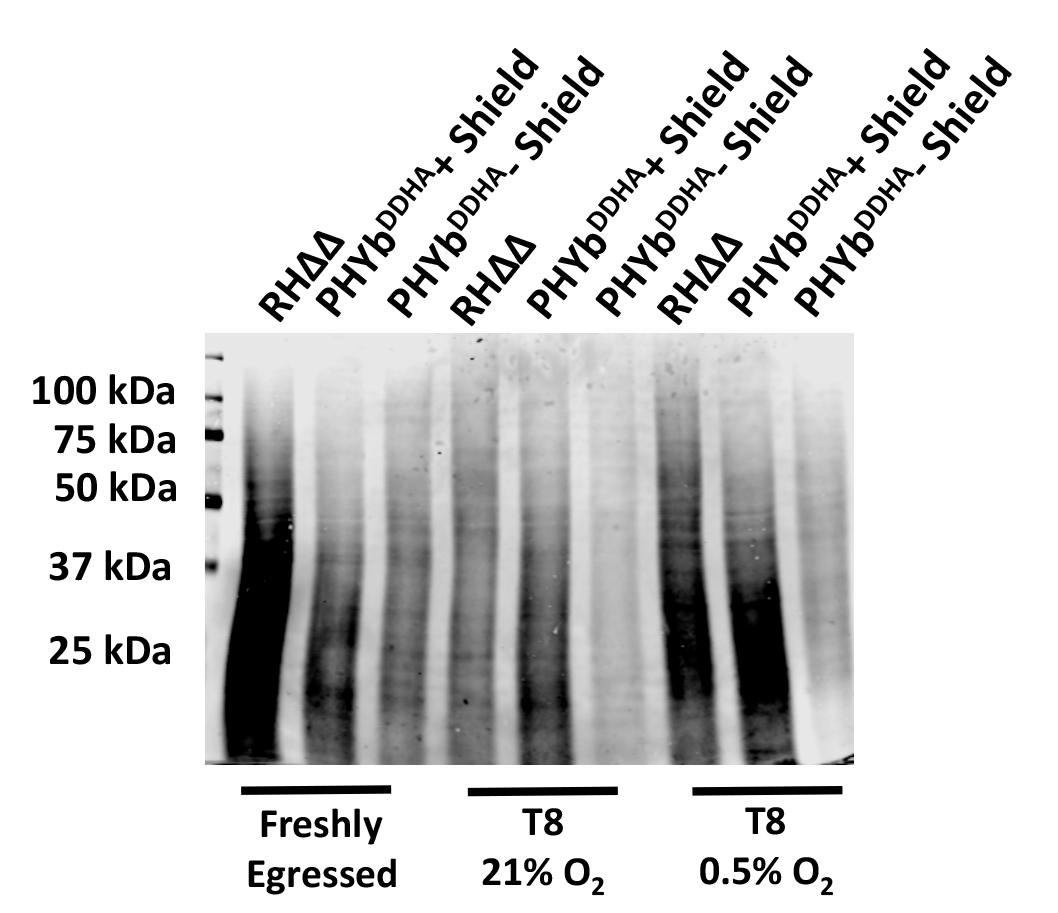

Supplement: FIG S7 [file mBio.00234-19-sf007.tif]

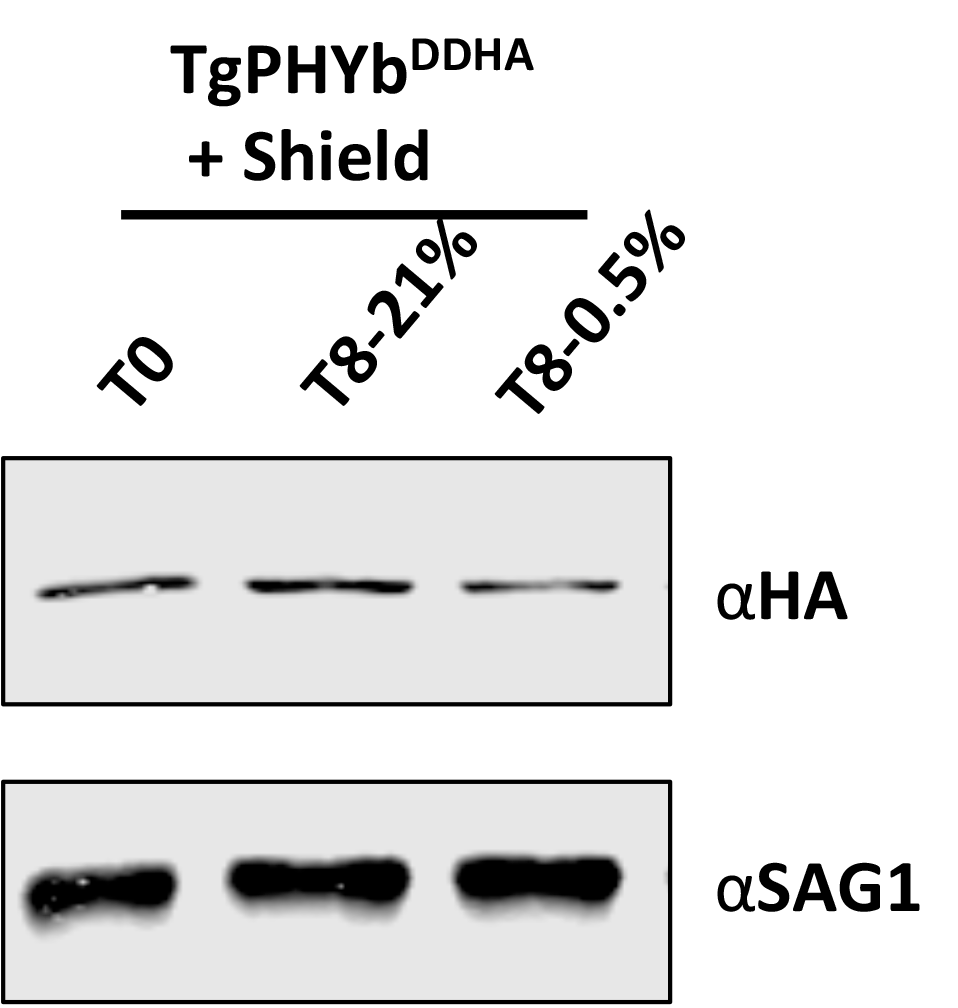

Supplement: FIG S8 [file mBio.00234-19-sf008.tif]
