# Supplementary material for: A Toxoplasma Prolyl Hydroxylase Mediates Oxygen Stress Responses by Regulating Translation Elongation
Source: mBio. 2019 Mar 26;10(2):e00234-19. doi: 10.1128/mBio.00234-19 (PMC6437050; doi:10.1128/mBio.00234-19)
Supplement: TABLE S1 [file mBio.00234-19-st001.pdf]

Supplemental Table S1

List of Antibodies:

Target protein	Source	Company	Catalog #
**eIF2α**	Rabbit	Gift from Dr. William J. Sullivan, Indiana University	
**P-eIF2α**	Rabbit	Gift from Dr. William J. Sullivan, Indiana University	
**eEF2**	Rabbit	Kerafast	ED7002
**P-eEF2**	Rabbit	Cell Signaling	#2331
**HA**	Rabbit	Cell Signaling	#3724
**Histone H3**	Mouse	Active Motif	#39763
**MIC2**	Mouse	Gift from Dr. Vern Carruthers, University of Michigan	
**SAG1**	Mouse	GenWay Biotech	#44A329
**SAG1**	Rabbit	Gift from Dr. John Boothroyd, Stanford University	
**Puromycin**	Mouse	Sigma-Aldrich	MABE343

List of Primers:

Target	Sequence
B1	**5'-GGAACTGCATCCGTTCATGAG-3'**<br>**5'-TCTTTAAAGCGTTCGTGGTC-3'**
mic2	**5′-GGGGTATGTGCTGTTGACG-3′**<br>**5'-GTGGCATTTCCGCAAGAC-3'**
phyB	**5'-TACTTCCAATCCAATTTAGCCCTGTATTGTCGTCCTTTTG-3'**<br>**5'-TCCTCCACTTCCAATTTTAGCAACGTCGTCCCCAGGACCG-3'**
rop18	**5′-GCAAACGCATCCTGGCGCAC-3′**<br>**5'-CCGAGTTGCCACGCGTCAGT-3'**
α-tubulin	**5′-ATGCCCTCTGACAAGACCATTGGA-3′**<br>**5'-GGCTCCAAATCCAAGAAGACGCAT-3'**
